# Integrating strategies of metabolomics, network pharmacology, and experiment validation to investigate the processing mechanism of Epimedium fried with suet oil to warm kidney and enhance yang

**DOI:** 10.3389/fphar.2023.1113213

**Published:** 2023-01-25

**Authors:** E. Sun, Ran Huang, Ke Ding, Ling Wang, Jian Hou, Xiaobin Tan, Yingjie Wei, Liang Feng, Xiaobin Jia

**Affiliations:** ^1^ The Third Clinical Medical College, Nanjing University of Chinese Medicine, Nanjing, China; ^2^ Key Laboratory of New Drug Delivery System of Chinese Materia Medica, Jiangsu Academy of Traditional Chinese Medicine, Nanjing, China; ^3^ Affiliated Hospital of Nanjing University of Chinese Medicine, Nanjing, China; ^4^ School of Traditional Chinese Pharmacy, China Pharmaceutical University, Nanjing, China

**Keywords:** Epimedium, metabolomics, network pharmacology, processing mechanism, kidney yang deficiency syndrome

## Abstract

**Introduction:** Epimedium, a traditional Chinese medicine (TCM) commonly used in ancient and modern China, is one of the traditional Chinese medicines clinically used to treat kidney yang deficiency syndrome (KYDS). There are differences in the efficacy of Epimedium before and after processing, and the effect of warming the kidney and enhancing yang is significantly enhanced after heating with suet oil. However, the active compounds, corresponding targets, metabolic pathways, and synergistic mechanism of frying Epimedium in suet oil to promote yang, remain unclear.

**Methods:** Herein, a strategy based on comprehensive GC-TOF/MS metabolomics and network pharmacology analysis was used to construct an “active compounds-targets-metabolic pathways” network to identify the active compounds, targets and metabolic pathways involved. Subsequently, the targets in kidney tissue were further validated by real-time quantitative polymerase chain reaction (RT-qPCR). Histopathological analysis with physical and biochemical parameters were performed.

**Results:** Fifteen biomarkers from urine and plasma, involving five known metabolic pathways related to kidney yang deficiency were screened. The network pharmacology results showed 37 active compounds (13 from Epimedium and 24 from suet oil), 159 targets, and 267 pathways with significant correlation. Importantly, integrated metabolomics and network pharmacologic analysis revealed 13 active compounds (nine from Epimedium and four from suet oil), 7 corresponding targets (ALDH2, ARG2, GSTA3, GSTM1, GSTM2, HPGDS, and NOS2), two metabolic pathways (glutathione metabolism, arginine and proline metabolism), and two biomarkers (Ornithine and 5-Oxoproline) associated with improved kidney yang deficiency by Epimedium fried with suet oil.

**Discussion:** These finds may elucidate the underlying mechanism of yang enhancement *via* kidney warming effects. Our study indicated that the mechanism of action mainly involved oxidative stress and amino acid metabolism. Here, we demonstrated the novel strategies of integrating metabolomics and network pharmacology in exploring of the mechanisms of traditional Chinese medicines.

## 1 Introduction

Epimedium, also known as Xianlingpi, is the dried leaves of a Berberis plant *Epimedium brevicornum* Maxim*., Epimedium sagittatum* (Sieb.et Zucc.) Maxim*., Epimedium pubescens* Maxim., or the dried leaves of *Epimedium koreanum* Nakai, which was first recorded in Shennong Materia Medica Classic and listed as a medium product, with functions of tonifying the liver and kidney, strengthening muscles and bones, and removing wind and dampness (Chinese Pharmacopoeia Commission [CPC], 2020). There are more than 20 processing methods for Epimedium, including stir-frying, wine roasting, salt roasting, ghee roasting, and suet oil roasting, among which suet oil roasting is the most commonly used and has been included in The Chinese Pharmacopoeia 2020 edition. According to traditional processing theory, suet oil is sweet and warm, possessing tonifying effects to treat deficiency syndromes. Epimedium fried with suet oil can play a synergistic role in strengthening its effect of warming the kidney and enriching the yang, mainly for impotence and infertility treatments. Clinical studies showed that Epimedium raw product focuses on dispelling rheumatism, and strengthening muscles and bones.

Kidney yang deficiency syndrome (KYDS) is a common syndrome type in Chinese medicine consultation, and it is also the basic syndrome type of various diseases. Insufficient kidney Yang may cause somnolence, cold, cold limbs, impotence, infertility in women, diarrhea, along with other symptoms of Yang deficiency (Wu et al., 2022). In the pharmacological research on kidney-yang deficiency, glucocorticoids, sex hormones, adenine, and kidney/bilateral adrenalectomy are often used to establish animal models of kidney-yang deficiency. Conventionally, they are supplemented with large doses of exogenous glucocorticoids (hydrocortisone) to further establish the model (Sun et al., 2017).

In previous studies, our research mainly focused on the mechanism of “heating” and “suet oil” processing factors from the perspective of the absorption and metabolism of flavonoids in Epimedium, indicating that there were differences in the absorption and metabolism of flavonoid glycosides in Epimedium (Chen et al., 2009). In the processing process, “heating” could change the content of main active flavonoids of Epimedium, hence produced more easily absorbed bioactive flavonoids (Chen et al., 2007; Sun et al., 2014). The excipient “suet oil” could further promote the formation of self-assembled micelles of Epimedium flavonoids *in vivo*, increased the solubility of active flavonoids, and improved the absorption of active flavonoids, to achieve the purpose of synergism (Gu et al., 2019). However, the effect of traditional Chinese medicine on human body is a biological process of interaction and integration between “intervention system (TCM) and response system (biological organism)" (Xiang et al., 2012). It is difficult to match corresponding disease targets through the disease database for TCM diseases, especially for deficiency diseases. Therefore, it is necessary to adopt new methods and strategies to systematically and comprehensively explore the processing mechanism of TCM.

Metabolomics can analyze all metabolites in the whole sample and comprehensively monitor the changes of metabolites in the body. Applying the methods and ideas of metabolomics to the processing of TCM can reveal the changes of metabolism pathway and action network of TCM before and after processing as a whole (Kui et al., 2022). Network pharmacology is a new method to systematically and integrally study drugs from the perspective of biological network stability. It can systematically study the molecular relationship between TCM components and complex diseases (Xie et al., 2019). Metabolomics can be used to construct the target database of disease metabolites, hence the combined application of metabolomics and network pharmacology is gradually increasing (Chen et al., 2019). The multi-omics technology in systems biology may elucidate the processing mechanism of TCM, from the active components to the target genes, proteins, metabolites and metabolic pathways of the body. Therefore, the integration of multi-omics technology is vital to clarify the mechanism of action in TCM and provide insights into the processing mechanism of Epimedium.

Epimedium treatment may cause the linkage effect of “active component group, target *in vivo* and biological metabolism”, so the changes of downstream biological metabolites directly reflect the status of upstream targets. Therefore, based on previous research, this study used metabonomics—Network pharmacology—Real-time quantitative polymerase chain reaction (RT qPCR) integration technology to obtain reasonable and objective data from biomarkers, metabolic pathways, active ingredient groups and potential targets, and build a “component—target—metabolism” network, thereby revealing the processing and synergistic mechanism of Epimedium fried with suet oil for warming the kidney and promoting yang on the whole. This study provides new insights for studying processing mechanism of traditional Chinese medicine.

## 2 Materials and methods

### 2.1 Reagents and materials

Epimedium was purchased from Shanxi, identified as *Epimedium brevicornum* Maxim., courtesy of Professor Baolin Guo, Institute of Pharmaceutical Botany, Chinese Academy of Medical Sciences (batch number: 161231). Hydrocortisone injections were bought from Tianjin King York Group Co., Ltd. (batch number: 1407271; Tianjin, China). Suet oil was purchased from Inner Mongolia Xilin Gol Grassland Co., Ltd. (Inner Mongolia, China). Formalin solution was purchased from Shanghai Aladdin Bio-Chem Technology Co., LTD. (batch number: F140859, Shanghai, China). Hydrochloric Acid was purchased from Nanjing Aojia Chemical Co., Ltd. (batch number: 2013050203, Nanjing, China). 2-Chloro-L-phenylalanine (content ≥98%) was purchased from Shanghai Hengbo Biological Technology Co., Ltd. (Shanghai, China). Bis(trimethylsilyl) trifluoroacetamide (99% BSTFA +1% TMCS) was provided by Regis Technologies, Inc. (Morton Grove, United States). Sodium azide was bought from Shandong Xiya Chemical Industry Co., Ltd. (batch number: S7470, Jinan, China). Urease was bought from Sigma-Aldrich (batch number: SLBB0100V, St. Louis, MO, United States). Pyridine was purchased from Adamas Reagent, Ltd. (batch number: 030928, Shanghai, China). Chromatography grade methanol was purchased from TEDIA (batch number: 1603253, State of Ohio, United States). Ethanol (analytically pure) was purchased from Nanjing Chemical Reagent Co., Ltd. (batch number: 1603233, Nanjing, China). Trichloromethane (batch number: 10006818) and propan-2-ol (batch number: 80109218) were purchased from Sinopharm Chemical Reagent Co., Ltd. (Shanghai, China). HyPure TMMolecular Biology Grade Water was provided by HyClone (batch number: SH30538.02, Logan, United States).

Corticosterone (CORT), Adrocorticotropin (ACTH), 17-Hydroxysteroids (17-OHCS), trifluorothyronine (T3), Thyroxine (T4), thyroid-stimulating hormone (TSH), luteinizing hormone (LH), testosterone (T), and follicle-stimulating hormone (FSH) kits were purchased from Nanjing Herbaceous Source Biotechnology Co., LTD.

Agilent 7890B gas chromatography system (Agilent, United States) coupled with a LECO Chroma TOF Pegasus HT mass spectrometer (LECO, United States) were employed for metabonomics analysis. Ultrapure water was prepared by a Milli-Q water purifier (Merck Millipore, Germany). Heraeus Fresco 17 Centrifuge (Thermo Scientific, United States). BPG-9050AH high temperature blast drying oven (HASUC, China). Vortex Genie^®^ 2 Vortex instrument (Scientific Industries, United States). CX23 electron microscope (Olympus, Japan). TNG-T98 Frozen Concentration Centrifugal Dryer (Taicang Huamei, China). ELISA (Spectra max 190, Shanghai Meigu Molecular Instrument Co., Ltd., shanghai, china). Quantitative Real-time PCR LightCycler instrument was provided by Bio-Rad Laboratories, Inc. (California, United States). Servicebio^®^ RT First Strand cDNA Synthesis Kit and 2×SYBR Green qPCR Master Mix (None ROX) were obtained from Wuhan servicebio technology Co., LTD. (Wuhan, China).

### 2.2 Preparation of Epimedium processing and extraction

The prepared Epimedium decoction pieces (1 kg) were selected and made three copies in parallel. One sample was evenly added with hot melted suet oil (200 g), and mixed well. Then, it was oven-baked at 170°C for 7 min to get the Epimedium fried with suet oil. The second sample was placed into the oven without oil to obtain a similarly heated Epimedium. The third decoction piece was reserved as raw control.

All three samples were immersed in boiling water (1:30) for 1 h before extraction. Then it was extracted three times, 30 min for each time, filtered through gauze, respectively. The next three filtrates were merged and concentrated under vacuum, thus extracting solution was obtained respectively. The extraction flow chart is shown in Supplementary Figure S1.

### 2.3 Animals and treatments

Thirty-six adult male Sprague-Dawley rats (body weight 230 ± 20 g, animal licence No. SCXK(HU)2014-0001) were provided by Animal Experiment Center of Soochow University (Suzhou, China), and raised in the Experimental Animal Center of Jiangsu Academy of Traditional Chinese Medicine (Nanjing, China). Rats were housed in a climate-controlled room (relative humidity of 45% and ambient temperature 22°C–24°C), kept on a 12 h/12 h light-dark cycle, with free access to food and drinking water.

After a 2-week habituation, all rats were randomly divided into 6 groups with 6 rats in each; Control group (N, No. N1∼N6), Model group (M, No. M1∼M6), Suet oil group (A, No. A1∼A6), Epimedium raw product group (B, No. B1∼B6), Epimedium heating product group (C, No. C1∼C6), Epimedium fried with suet oil group (D, No. D1∼D6). M, A, B, C, and D group rats were injected intraperitoneally with hydrocortisone once daily for the following 10 days. The injection dose was 15 mg kg^−1^ on the first day, the dose was halved to 7.5 mg kg^−1^ on the second day and subsequent 9 days. N group was injected intraperitoneally with an equal volume of 0.9% sodium chloride injection. A, B, C, and D group rats were administered *via* intragastric with suet oil emulsion (0.144 g kg^−1^, 20% of Epimedium content, dispersed in water with CMC-Na), the Epimedium raw product extract (0.72 g kg^−1^), the Epimedium heating product extract (0.72 g kg^−1^), and the Epimedium fried with suet oil extract (0.72 g kg^−1^), respectively. N and M groups were orally administered with the same volume of pure water. The intragastric administration was performed once a day for 10 days. Meanwhile, body weights of the rats in each group were recorded daily. All animal treatments and experiments were approved by the Animal Ethics Committee of Jiangsu Provincial Academy of Chinese Medicine, and were strictly performed in accordance with the National Institutes of Health Guide for the Care and Use of Laboratory Animals (AEWC-20200702-119).

### 2.4 Collection of samples

After the 10th day of administration, all rats were fasted but had free access to water for 12 h while urine samples were collected. The 12 h urines of all rats were collected into a 50 mL centrifuge tube containing 20 µL of 1% sodium azide (NaN_3_). After centrifugation at 4,500 rpm min^−1^ for 10 min in a 4°C low-temperature centrifuge, the supernatant was taken and stored at −80°C until metabonomics analysis. Afterwards, blood samples were drawn from the orbit and collected in two 2 mL centrifuge tubes, one of which contained 20 µL heparin sodium solution for preparing plasma samples, the other for preparing serum samples. Then, centrifugation was performed at 4,000 rpm min^−1^ for 10 min in a 4°C low-temperature centrifuge, and the supernatant was taken to obtain plasma samples and serum samples. The plasma and serum samples were stored at −80°C until plasma metabonomics analysis and serum biochemical analysis.

Biochemical indicators included the hypothalamus-pituitary-adrenal axis in kidney-yang deficiency rats (Chen and Wang, 2015; Liu et al., 2016; Zhang et al., 2020): CORT, ACTH and 17-OHCS; Hypothalamus-pituitary-thyroid axis related indicators: T3, T4, and TSH; Hypothalamus-pituitary-gonad axis related indicators: LH, T, and FSH.

Thereafter, all rats were euthanized in parallel. The kidney tissues were quickly excised, directly frozen in liquid nitrogen, and stored at −80°C for subsequent quantitative real-time PCR assays. Simultaneously, the hypothalamus, pituitary, thyroid, adrenal gland, testis and kidney of all rats were collected and fixed in formalin solution for histopathological analysis.

### 2.5 Preparation of metabonomic samples

The urine samples were thawed before analysis and 100 µL aliquots of urine sample were added to 20 µL urease (80 mg mL^−1^). Then the mixture was shaken and mixed for 30 s, and incubated in an oven at 37°C for 1 h. Subsequently, urine sample mixture and 100 µL aliquots of plasma sample were mixed with 350 µL methanol and 20 µL L-2-chlorophenylalanine. Post vortex-mixing for 30 s and centrifugation at 16000 rpm for 20 min at 4°C, the supernatant of urine sample and plasma sample were transferred into a 2 mL gas sample bottle (siliconized methane). Moreover, 11 µL of each urine sample and plasma sample were accurately pipetted and mixed as the urine and plasma quality control (QC) sample, respectively. After the supernatants of the urine sample and plasma samples were completely dry in a vacuum concentrator, 60 µL of methoxyamine salt reagent (methoxamine hydrochloride, dissolved in 20 mg mL^−1^ pyridine) was added and mixed well, and incubated in an oven at 80°C for 30 min. Thereafter, 80 µL of BSTFA (containing 1% TCMS, v/v) were quickly added to each sample. The mixture was incubated in an oven at 70°C for 2 h and cooled to 25°C. Finally, 10 µL saturated fatty acid methyl ester standard mixture was added and mixed well for GC-MS analysis.

### 2.6 Metabonomics analysis

The analysis of plasma and urine samples was performed on an Agilent 7890B gas chromatography system (Agilent, United States) coupled with a LECO Chroma TOF Pegasus HT mass spectrometer (LECO, United States), which was equipped with Agilent DB-5MS capillary column (30 m × 0.25 mm × 0.25 μm, J&W Scientific, Folsom, CA, United States). GC-TOF-MS analysis conditions were set as follows: the injection volume was 1 µL with non-split mode. Carrier gas was helium used with a column flow rate of 1.0 mL min^−1^, the column temperature maintained at 50°C for 1 min and then heated at a rate of 20°C·min^−1^ up to 310°C maintained for 6 min. The forward inlet purge flow rate was 3 mL min^−1^ and the forward inlet temperature was 280°C. Transmission line temperature and ion source temperature were 270°C and 220°C respectively. The ionization voltage was −70 eV. The scan range and speed were 50–500 m/z, 20 spectra/sec. The solvent delays of urine and plasma samples were 455 S and 366 S, respectively.

Random urine samples (N2), plasma samples (N5) and the internal standard sample (L-2-chlorophenylalanine) were tested according to the above conditions. Thereafter, a comparative analysis of the GC-TOF-MS total ion characteristics (TIC) between the random urine sample (N2), plasma samples (N5) and the internal standard sample were analyzed in order to corroborate and validate our experimental methodologies as well as the instrument platforms. The 45 urine samples (including 9 urine QC samples) and plasma samples (including 9 plasma QC samples) were also tested according to the above conditions, and TIC of urine and plasma GC-TOF-MS were obtained for all samples. The retention time of the internal standard (L-2-chlorophenylalanine) in each TIC was extracted to investigate the stability of the system.

First, the raw data collected by the GC-TOFMS system were preprocessed. Single data and single peak in the original data were filtered, the missing value in the original data were recoded, and the internal standard (IS) to were utilized to normalize each group of data. Meanwhile, both TOF 4.3X software (LECO Corporation, United States) and LECO Fiehn Rtx5 database were used for raw peak exact matching, baseline filtering and calibration, peak alignment, deconvolution analysis, peak identification and integration of peak area. Finally, the standardized data were imported into SIMCA software (V14.1, MKS Data Analytics Solutions, Umea, Sweden) for multivariate statistical analysis, such as, principal component analysis (PCA) and orthogonal projection to latent structures-discriminant analysis (OPLS-DA) analysis. Prior to PCA, all variables obtained from GC-MS data sets went through logarithmic (LOG) transformation and centralization (CTR) scaling. Meanwhile, prior to OPLS-DA, all variables obtained from GC-MS data sets underwent logarithmic (LOG) transformation and unit variance (UV) scaling. Afterwards, the validity of the OPLS-DA model was tested by 7-fold cross validation and permutation test (random 200 times). Multivariate statistical analysis was conducted, combined with traditional univariate statistical analysis, with variable importance in the projection of the first principal component of the OPLS-DA model greater than 1 (VIP >1), while the *p*-value of Student’s t-test is less than 0.05 (*p* < 0.05), was used to screen significantly different metabolites, and the result was visualized in the form of a volcano graph. Thereafter, significantly different metabolites were subjected to pathway analysis with KEGG (https://www.kegg.jp/) and MetaboAnalyst 5.0 (https://www.metaboanalyst.ca/), which is a web-based tool for visualization of metabolomics, to identify related metabolic pathways. Metabolic pathways with *p* < 0.05 or Impact >0.10 were screened as the most relevant metabolic pathways and may be used to distinguish the kidney-yang deficiency model group from the normal control group.

### 2.7 Network pharmacology

The active ingredients of Epimedium fried with suet oil mainly include two parts: the active ingredients of Epimedium flavonoids and the chemical ingredients of “suet oil” as the processing auxiliary material. Based on the previous research of the research group (Jiang et al., 2014; Li et al., 2020), 32 flavonoids were identified in raw and processed Epimedium products and 25 fatty acids were identified in suet oil, and made available *via* PubChem database (https://pubchem.ncbi.nlm.nih.gov/) to be downloaded in SDF format for the 2D or 3D structure of each component. The components were then screened with a definite 2D or 3D structure as the component library of Epimedium fried with suet oil. Thereafter, based on the PharmMapper database (http://www.lilab-ecust.cn/pharmmapper/), the species was set to “Homo Sapiens”, and the potential targets of Epimedium pilaris were predicted. According to the z score value, the top 15 targets of each component corresponding to the score were screened as the action targets of chemical components in Epimedium. Targets were canonicalized to standard gene names *via* the UniProt database (https://www.uniprot.org). The targets of chemical constituents in Epimedium fried with suet oil are imported into the STRING database (https://www.string-db.org/) to construct a PPI network, and then the targets are imported into Cytoscape 3.6.1 to construct an active ingredient-potential target network. Finally, these targets were uploaded to the Metascape database (https://metascape.org/gp/index) for KEGG pathway analysis.

### 2.8 Integrated metabonomics and network pharmacology analysis

The metabolic pathways of *p* < 0.05 or Impact >0.1 obtained from the metabonomics analysis were mapped to pathways acquired by the network pharmacology to uncover pathways in which the two correlates. Afterwards, target proteins and active compounds corresponding to Epimedium fried with suet oil from network pharmacology were reversely searched from the overlapping pathways, and finally the potential active compounds, target proteins, and metabolic pathways of Epimedium fried with suet oil affecting KYDS were obtained. On this basis, these potential targets were further verified *via* RT-qPCR, and differential target proteins were shown.

### 2.9 Quantitative real-time PCR analysis

Firstly, total RNA was isolated and extracted from flash-frozen kidney tissues. 100 mg of tissue was taken and added into a pre-cooled homogenized tube (containing 1 mL RNA extract). Grind thoroughly with the grinder until no tissue is visible, and then take the supernatant after centrifuging 10 min at 12,000 rpm. Then 250 µL of trichloromethane was added, the centrifuge tube was inverted for 15 s, mixed well, and left for 3 min. After separating the cores at 12,000 rpm at 4°C for 10 min, 400 µL of supernatant was transfered to a new centrifugal tube, adding 0.8 times the volume of propan-2-ol and mixing it upside-down. After being placed at −20°C for 15 min and centrifuged at 12,000°C for another 10 min, the white precipitate at the bottom of the tube was RNA. The liquid was removed by suction, and 1.5 mL of 75% ethanol was added to wash the precipitate, and then centrifuged at 4°C at 12,000 rpm for 5 min. After removing the liquid by suction, the centrifuge tube was placed on the ultra-clean table and blown for 3 min. The RNA was dissolved by adding 15 µL of RNase-free water and incubated at 55°C for 5 min. The concentration and purity of total RNA samples were determined by Nanodrop 2000. Thereafter, RNA was reversely transcribed to cDNA by using Servicebio^®^RT Enzyme Mix. Finally, RT-qPCR and data collection were performed with PCR Master Mix (Bio-rad). The samples were exposed to pre-denaturation at 95°C for 10 min, followed by 40 cycles of denaturation at 95°C for 15 s and annealing at 60°C for 30 s. The dissolution curve conditions were from 65°C to 95°C, and the fluorescence signal was collected for every 0.5°C increase in temperature. Target mRNA expression in each sample was normalized to the housekeeping gene (GAPDH) to normalize the starting cDNA levels. The 2^−ΔΔCT^ method was used to calculate relative mRNA expression levels. The PCR primers were listed in Supplementary Table S1. The experiment was repeated thrice.

### 2.10 Statistical analysis

The data from body weight and biochemical indicators were screened by SPSS 26.0 statistical software package (IBM, Armonk, NY, United States) and GraphPad Prism 6.01 (GraphPad Software, Inc., San Diego, CA, United States) software with Student’s t-test or one-way analysis of variance (ANOVA). Data were presented as the mean ± standard deviation. In all experiments, confidence level was set at 95% and 99% to determine the significance of difference (*p* < 0.05, *p* < 0.01).

## 3 Results

### 3.1 Weight changes

**FIGURE 1:**
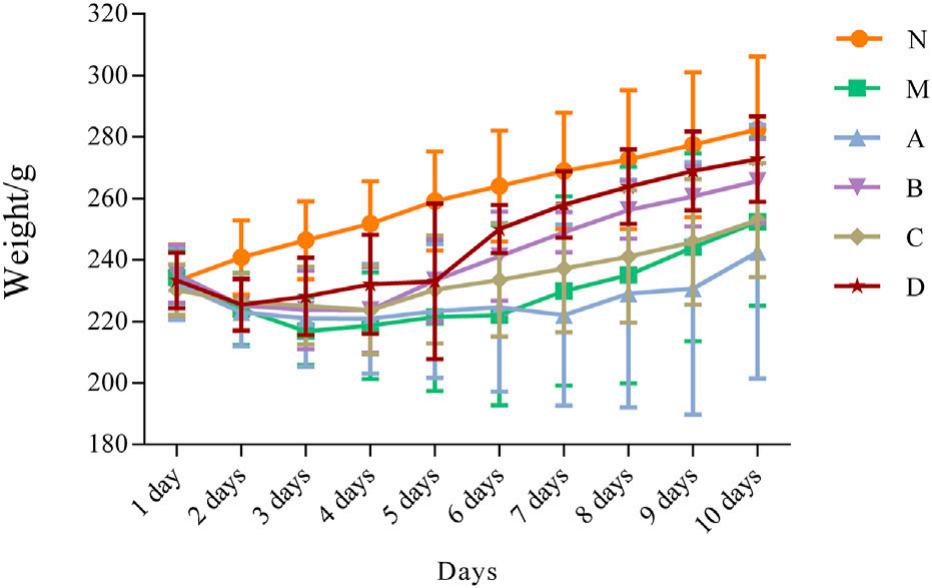
Line chart of changes in body weight of rats during the experiment N, normal group; M, model group; A, suet oil group; B, Epimedium raw product group; C, Epimedium heating product group; D, Epimedium fried with suet oil group.

The weight changes of rats in each group were shown in Figure 1, indicating gradual increase of the body weight of rats in the N group from day 1 until day 10 of the experiment. After modeling, the body weight of rats in the M group decreased to the lowest at day 3, and gradually recovered to the original body weight at day 10. The weight of rats in all treatment groups showed initial decrease followed by a gradual recovery, and the weight changes of rats in the D group was approximate to that of N group.

### 3.2 Biochemical and histopathological results

**FIGURE 2:**
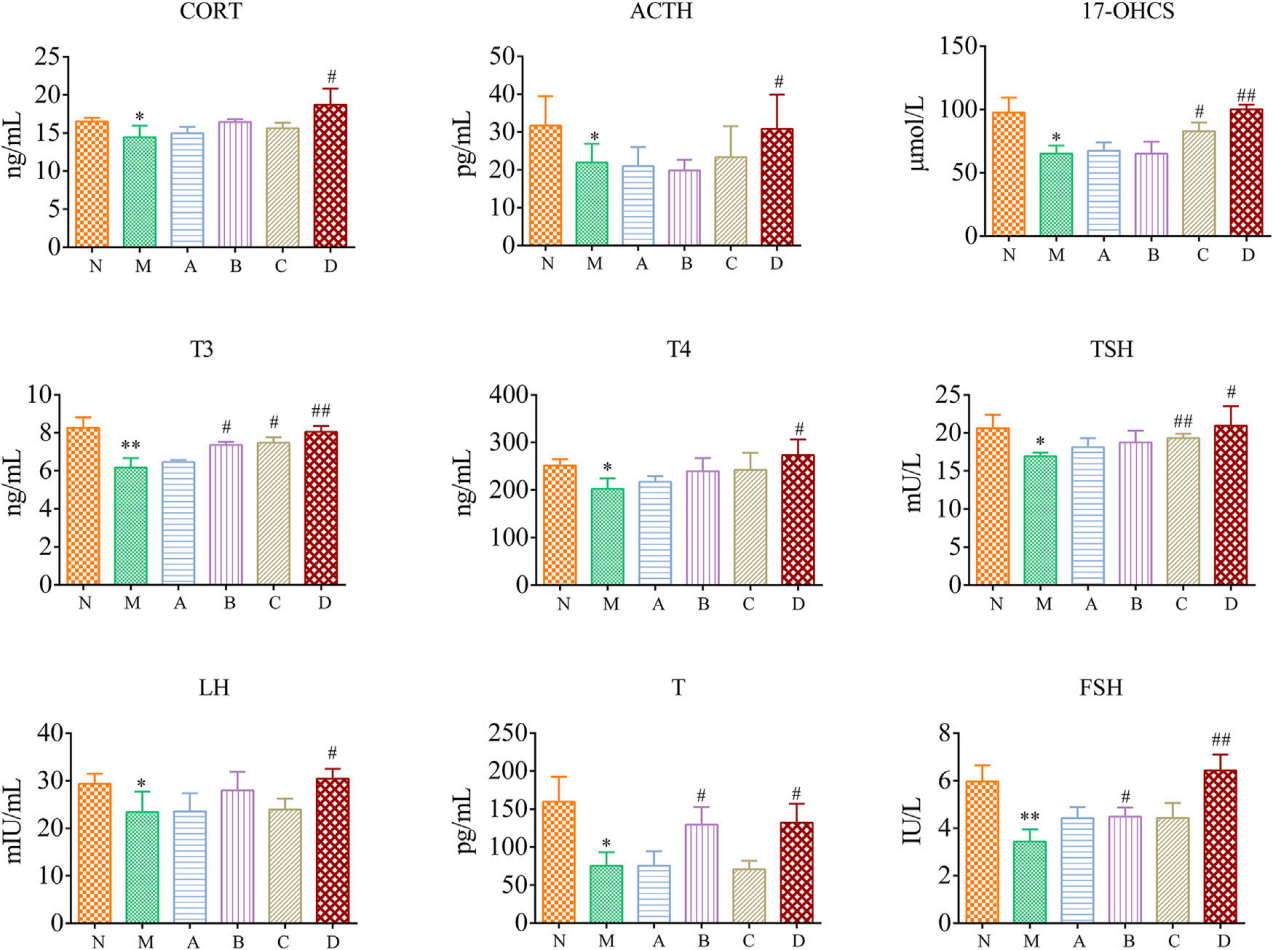
Biochemical indexes in kidney-yang deficiency rats N, normal group; M, model group; A, suet oil group; B, Epimedium raw product group; C, Epimedium heating product group; D, Epimedium fried with suet oil group. Significance compared to N group, ^*^ *p* < 0.05, ^**^ *p* < 0.01; Significance compared to M group, ^#^ *p* < 0.05, ^##^ *p* < 0.01.

Figure 2 showed that compared with the N group, the levels of CORT, ACTH, 17-OHCS, T3, T4, TSH, LH, T, and FSH in the M group were significantly decreased (*p* < 0.05, *p* < 0.01). Apart from ACTH, the A group had a callback effect on other indicators, although there was no significant difference. The B group has significant callback to T3, T and FSH (*p* < 0.05). The C group had significant callback to 17-OHCS, T3, TSH (*p* < 0.05, *p* < 0.01). All biochemical indexes were significantly increased in the D group.

**FIGURE 3:**
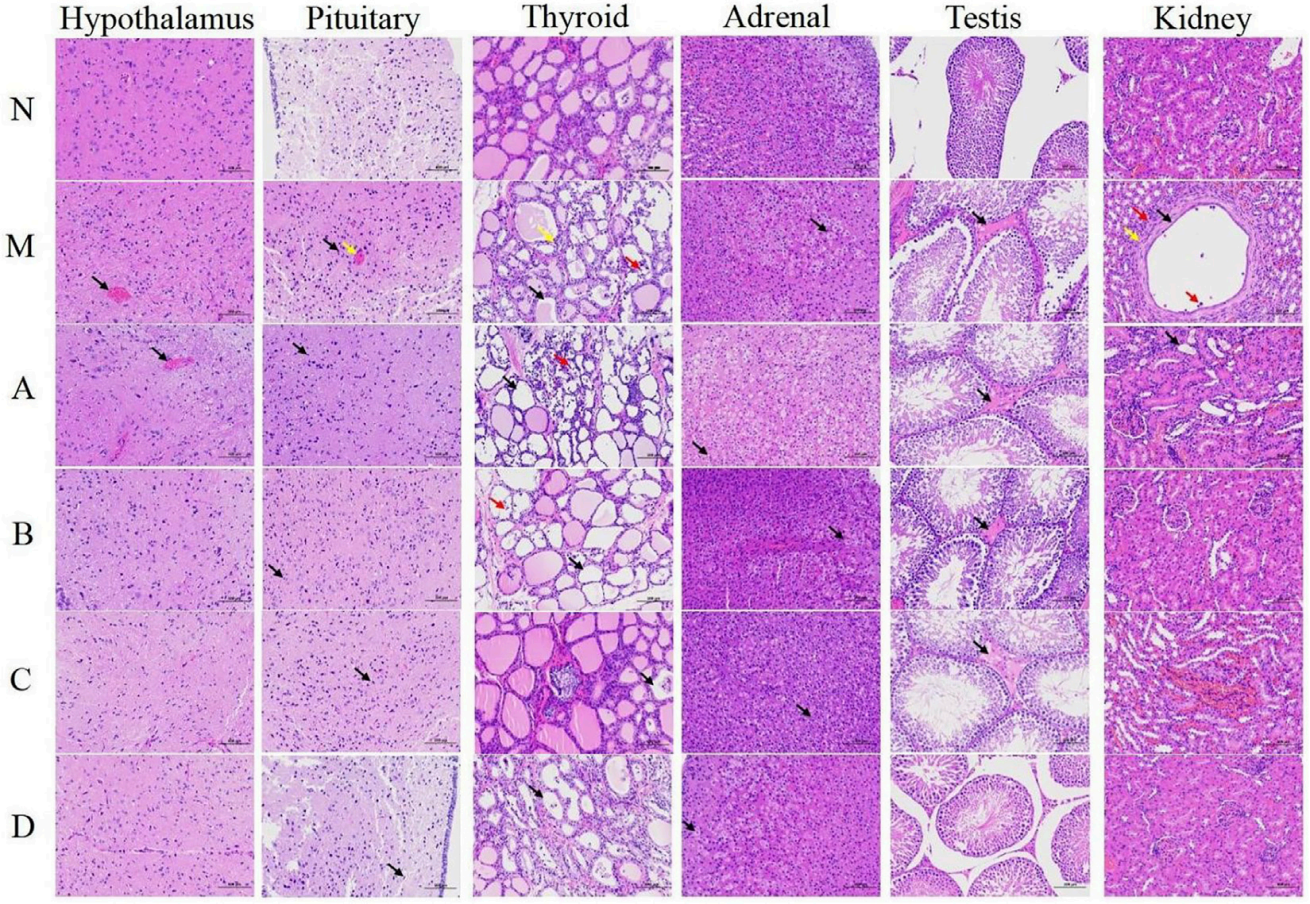
H&E staining of hypothalamus, pituitary, thyroid, adrenal, testis and kidney tissue sections (magnification ×200). N, normal group; M, model group; A, suet oil group; B, Epimedium raw product group; C, Epimedium heating product group; D, Epimedium fried with suet oil group. Hypothalamus: Black arrow represents the bleeding. Pituitary: Yellow arrow represents the bleeding. Black arrow represents the cell nucleus and the cytosolic boundary. Thyroid: Black arrow represents the follicular matrix. Yellow arrow represents epithelial cell necrosis. Red arrow represents nuclear fragmentation. Adrenal: Black arrow represents parenchymal cell degeneration. Testis: Black arrow represents eosinophilic serous material. Kidney: Black arrow represents the renal tubules. Yellow arrow represents the connective tissue. Red arrow represents the cellular infiltration.

As shown in Figure 3, in the kidney-yang deficiency group M, multiple mild hemorrhages were observed in the hypothalamus tissue. Pituitary tissue was slightly bleeding, the boundary between nucleus and cytoplasm was unclear. In the thyroid tissue, a large number of follicular matrix was reduced, many epithelial cells were necrotic and exfoliated, and nuclear fragmentation was observed. Hemorrhage was observed locally in adrenal tissue, a large number of parenchymal cells had granular degeneration, cells were swollen, and the cytoplasm was loose and lightly stained, showing fine granularity. A small amount of eosinophilic serous material exuded from multiple interstitial areas of testis tissue. In the renal medulla, the renal tubules were significantly dilated, accompanied by mild connective tissue hyperplasia, and punctate infiltration of lymphocytes and macrophages. These were consistent with the results of Tang et al. (Xiang et al., 2012). Except for group A, the hypothalamus-pituitary-target gland axis (hypothalamus, pituitary, thyroid, adrenal gland, testis) in group B/C/D was improved to a certain extent after administration, and the improvement effect of group D was better than that of group B/C. Taken together, these results suggested that the hypothalamic, pituitary, thyroid, adrenal, testis and kidney tissue and cell structures could be altered in the kidney-yang deficiency induced by intraperitoneal injection of hydrocortisone, again demonstrating that the hypothalamic-pituitary gland—the target gland axis was inhibited. After the intervention treatment of Epimedium processed by heating and suet oil, the pathological changes of tissue cell structure caused by kidney yang deficiency was shown to have improved, which further revealed the mechanism of Epimedium fried with suet oil to warm the kidney and promote yang.

### 3.3 Metabonomics results

#### 3.3.1 Metabolic profile analysis of urine and plasma in rats

The internal standard (L-2-chlorophenylalanine) retention time, the GC-TOFMS TIC of the random sample (N2, N5) and internal standard (L-2-chlorophenylalanine) were shown in Supplementary Tables S2, S3, Supplementary Figures S2–S5. The results showed that the experimental method was viable; the instrument platform was justified, and corroborated with the conditions in the experimental methodologies. Six groups of total ion current diagrams of the urine samples and serum samples analyzed by GC-MS were shown in Supplementary Figures S6, S7. A total of 759 active peaks were detected in urine and 261 active peaks in plasma.

#### 3.3.2 Multivariate statistical analysis and potential biomarkers exploring

**FIGURE 4:**
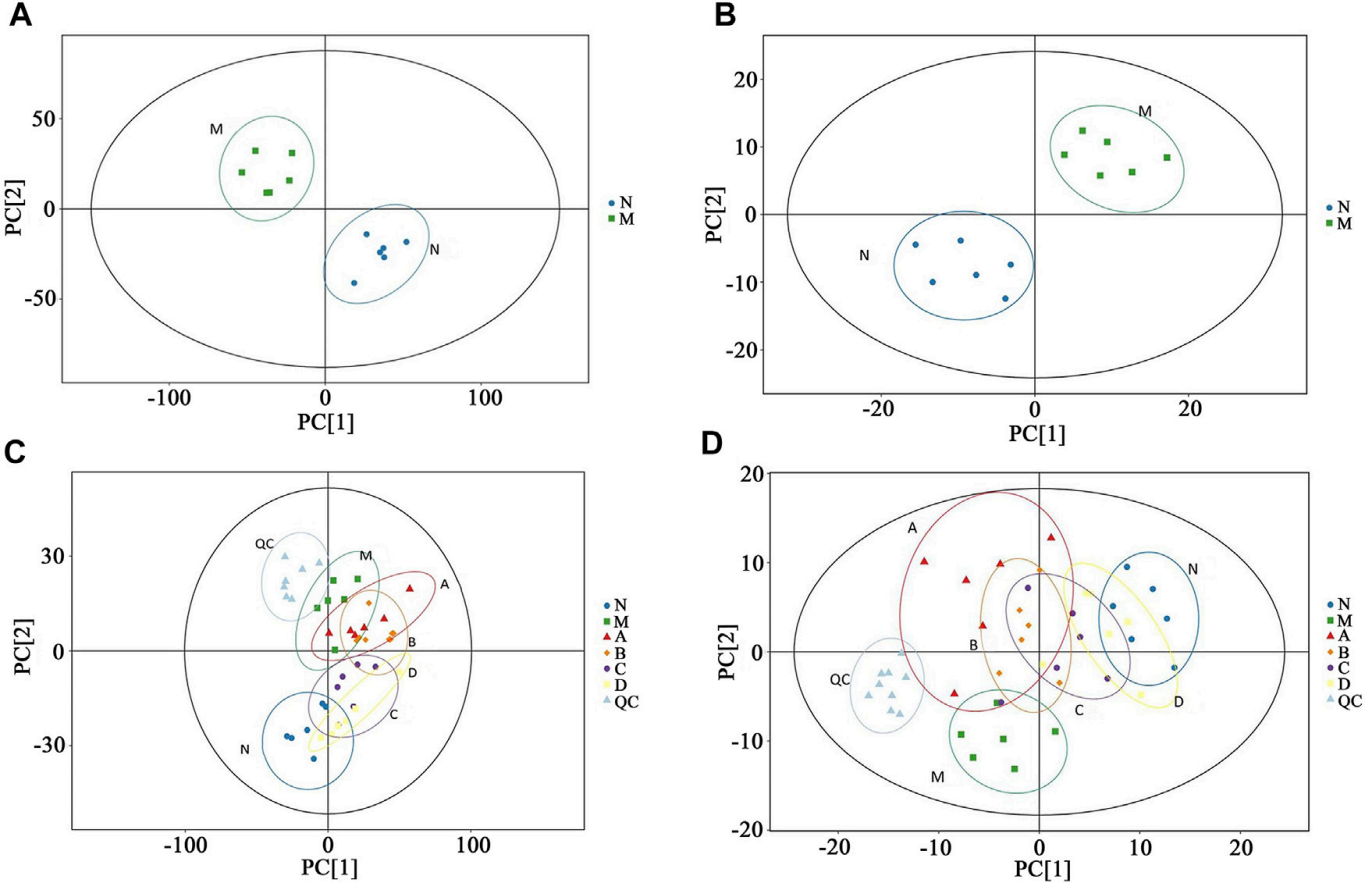
PCA scores plots of urine and plasma samples in rats N: Control group; M: Model group; A: Suet oil group; B: Epimedium raw product group; C: Epimedium heating product group; D: Epimedium fried with suet oil group. (N VS*.* M, **(A)** Urine, **(B)** Plasma; the PCA scores plots of six group, **(C)** Urine, **(D)** Plasma.)

An unsupervised PCA was performed first to characterize between-group differences in rat urine and plasma samples, respectively. As displayed in Figure 4A, B, the urine and plasma samples of the rats in the control group and the model group were clustered into two categories on the PCA score plots, indicating that after modeling, the urine and plasma endogenous metabolites of the rats in the two groups existed significant differences. As shown in Figures 4C, D, the rat urine and plasma QC samples were well-aggregated, indicating that the detection system was stable and reliable, and the obtained differences could reflect the biological differences between samples. The urine and plasma samples of the A group, the B group, the C group, and the D group were all separated from the M group in the PCA score plots and approached the N group, indicating that each administration group could regulate the metabolic disorder of hydrocortisone-induced kidney-yang deficiency rats to normal to different degrees, and the regulation trend was as follows: D group > C group > B group > A group.

**FIGURE 5:**
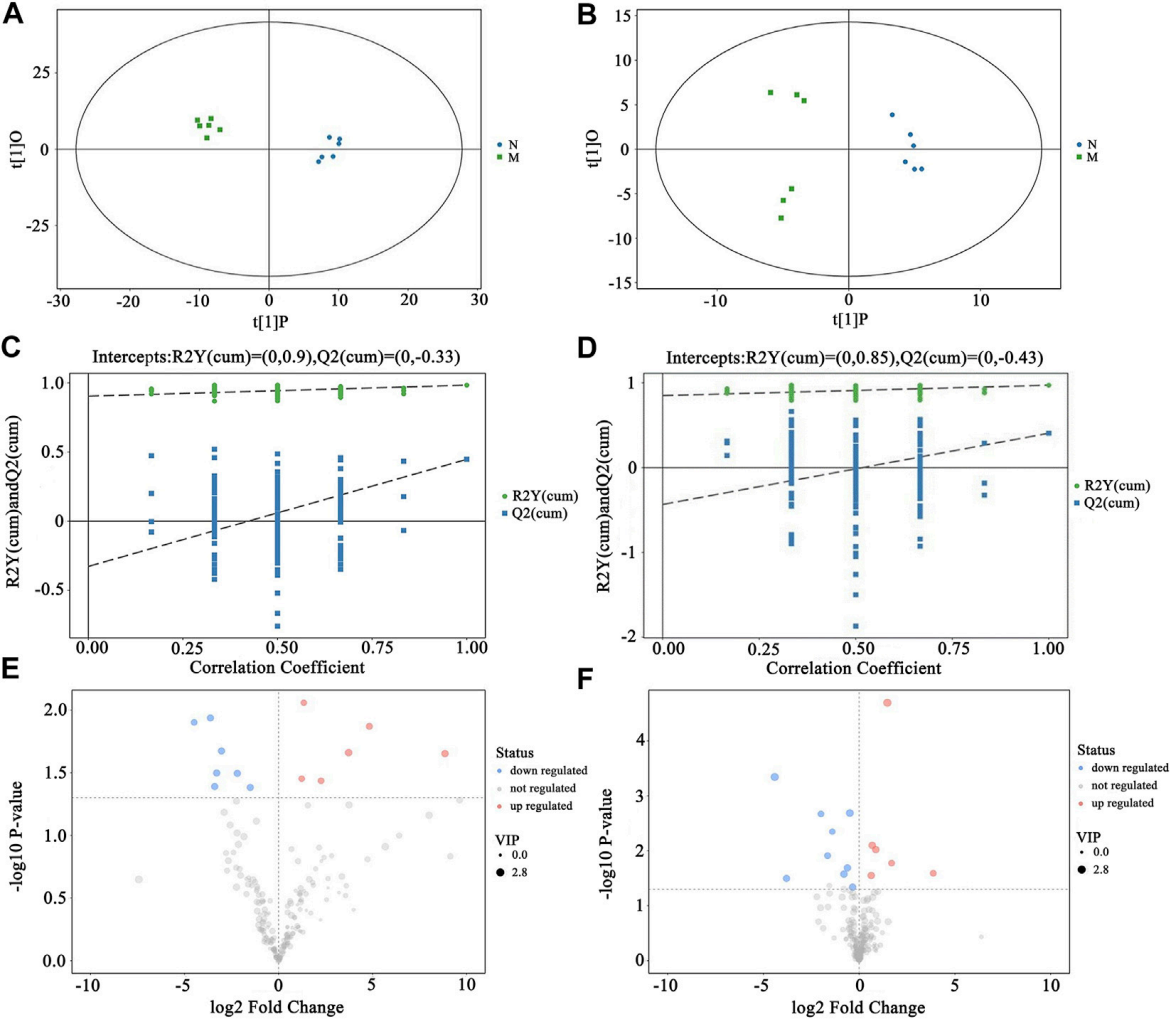
Screening of biomarkers in rats with kidney-yang deficiency. N VS*.* M, **(A)** Urine OPLS-DA scores plots, **(B)** Plasma OPLS-DA scores plots, **(C)** Urine OPLS-DA permutation test, **(D)** Plasma OPLS-DA permutation test, **(E)** Urine Volcano plot, **(F)** Plasma Volcano plot. Red represents upregulation of differential metabolites. Blue represents downregulation of differential metabolites. Gray represents metabolites with no statistical difference.

To further determine the differences of endogenous metabolites in the urine and plasma of rats after administration, OPLS-DA analysis was performed on the M group and the N group, respectively. As shown in Figure 5A, B, both in the urine samples and in the plasma samples, the N group and the M group were respectively clustered into one category in the OPLS-DA scores plots, indicating a significant difference between the N group and the M group. Then, a random permutation test (n = 200) was performed under the established OPLS-DA model to evaluate the model’s interpretation rate (*R* ^*2*^Y), predictive ability (*Q* ^*2*^) and other parameters. The results were arranged in the experiment. *R* ^*2*^ = 0.900, *Q* ^*2*^ = −0.330 (urine, Figure 5C); *R* ^*2*^ = 0.850, *Q* ^*2*^ = −0.430 (plasma, Figure 5D). The results show that the model has good stability and prediction ability, and no overfitting occurs. Combined with VIP >1 and *p* < 0.05 in the Volcano plot (Figures 5E, F), the differential variables with the largest contribution were screened as biomarkers related to kidney-yang deficiency syndrome, and were identified by LECO Fiehn Rtx5 database and combined with HMDB databases. Twenty-eight relevant biomarkers (13 in urine and 15 in plasma) were finally screened, of which 15 were identified (5 in urine and 10 in plasma). The results are listed in Table 1. Compared with the N group, nine biomarkers were upregulated in the M group, four of which were Tyramine, Galacturonic acid, Gulonate, 3-Hydroxyproline in urine. And 5 in plasma were *D*-Mannose, Ornithine, Sucrose, 2,4,6-trimethylPyridine, Cholesterol. Six biomarkers in the M group were downregulated, 1 in urine was downregulated as Ribitol, and five in plasma were downregulated as 5-Oxoproline, Citric acid, *L*-Tryptophan, Glycerol, Threonic acid.

**TABLE 1:** Biomarkers of urine and plasma in kidney-yang deficiency rats (n = 6, Mean ± SD).

Sample	Metabolite	*t*_R_/min	Formulas	Similarity	Datebase ID	VIP	*P*	Variation between groups				
								M vs*.* N	A vs*.* M	B vs*.* M	C vs*.* M	D vs*.* M
Urine	Ribitol	17.776	C_5_H_12_O_5_	864	HMDB0000508	2.103	0.022	↓^*^	↑	↑	↑	↑
Urine	Tyramine	22.732	C_8_H_11_NO	920	HMDB0000306	1.990	0.032	↑^*^	↑	↓	↓	↓
Urine	Galacturonic acid	23.108	C_6_H_10_O_7_	791	HMDB0002545	1.250	0.009	↑^**^	↓	↓	↓	↓
Urine	Gulonate	29.778	C_6_H_12_O_7_	909	HMDB0003290	2.468	0.022	↑^*^	↓	↓	↓	↓
Urine	3-Hydroxyproline	26.867	C_5_H_9_NO_3_	986	HMDB0245903	1.478	0.037	↑^*^	↓	↓	↓	↓
Plasma	5-Oxoproline	10.037	C_5_H_7_NO_3_	954	HMDB0000267	2.122	0.042	↓^*^	↑	↑	↑	↑
Plasma	*D*-Mannose	11.970	C_6_H_12_O_6_	942	HMDB0000169	2.097	0.046	↑^*^	↑	↓	↓	↓
Plasma	Ornithine	11.657	C_5_H_12_N_2_O_2_	935	HMDB0000214	2.202	0.027	↑^*^	↓	↓	↓	↓
Plasma	Citric acid	11.616	C_6_H_8_O_7_	916	HMDB0000094	1.918	0.021	↓^*^	↓	↑	↑	↑
Plasma	*L*-Tryptophan	13.690	C_11_H_12_N_2_O_2_	762	HMDB0000929	2.185	0.008	↓^**^	↑	↑	↑	↑
Plasma	Sucrose	15.340	C_12_H_22_O_11_	733	HMDB0000258	2.768	0.000	↑^**^	↓	↓	↓	↓
Plasma	2,4,6-trimethylPyridine	6.477	C_8_H_11_N	895	HMDB0245482	1.642	0.012	↑^*^	↓	↓	↓	↓
Plasma	Glycerol	8.229	C_3_H_8_O_3_	779	HMDB0000131	2.018	0.020	↓^*^	↑	↑	↑	↑
Plasma	Threonic acid	10.115	C_4_H_8_O_5_	745	HMDB0000943	2.184	0.010	↓^*^	↓	↑	↑	↑
Plasma	Cholesterol	19.184	C_27_H_46_O	872	HMDB0000067	2.427	0.002	↑^**^	↓	↓	↓	↓

‘↑’ and ‘↓’ represent compounds which are up- and downregulated in the M group compared with the N group or in the administration group (A/B/C/D) compared with the M group. *p < 0.05, **p < 0.01 (one-way ANOVA, with a Bonferroni correction). Similarity: Score the match between the substance and the peak detected by mass spectrometry.

#### 3.3.3 Regulation of differential metabolites

**FIGURE 6:**
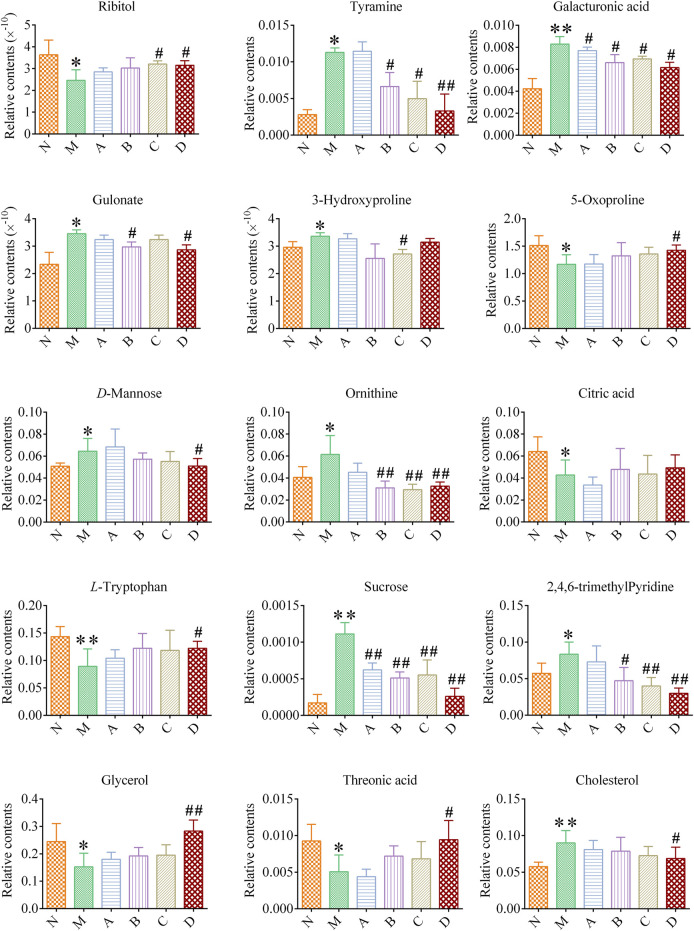
The levels of potential biomarkers of kidney-yang deficiency in each group Compared with the N group, ^*^ *p* < 0.05, ^**^ *p* < 0.01; compared with the M group, ^#^ *p* < 0.05, ^##^ *p* < 0.01.

Column charts were drawn according to the relative contents and change trends of the biomarkers in urine and plasma of rats in each group (Figure 6). After the treatment intervention, the B, C, and D group biomarker contents demonstrated certain normalizing trends, with that of the D group being the most significant. The Sucrose and Galacturonic acid could be significantly recalled in the A group (*p* < 0.05, *p* < 0.01), suggesting that the suet oil had some intervention effect on kidney-yang deficiency syndrome. The B group could significantly recall six differential metabolites, especially Ornithine and Sucrose (*p* < 0.01). The C group could significantly recall seven differential metabolites, especially Sucrose and 2,4,6-trimethyl-Pyridine (*p* < 0.01). The D group could significantly recall 13 differential metabolites, especially Tyramine, Ornithine, Sucrose, 2,4,6-trimethyl-Pyridine and Glycerol (*p* < 0.01). Compared with B and C groups, D group had the most differentmetabolites in callbacks, indicating that the effect of Epimedium fried with suet oil group was the best. It is possible that Epimedium fried with suet oil regulated kidney-yang deficiency in rats through the above-described metabolites, thereby enhancing the warming effect on the kidneys and strengthen yang.

#### 3.3.4 Metabolic pathway analysis

**FIGURE 7:**
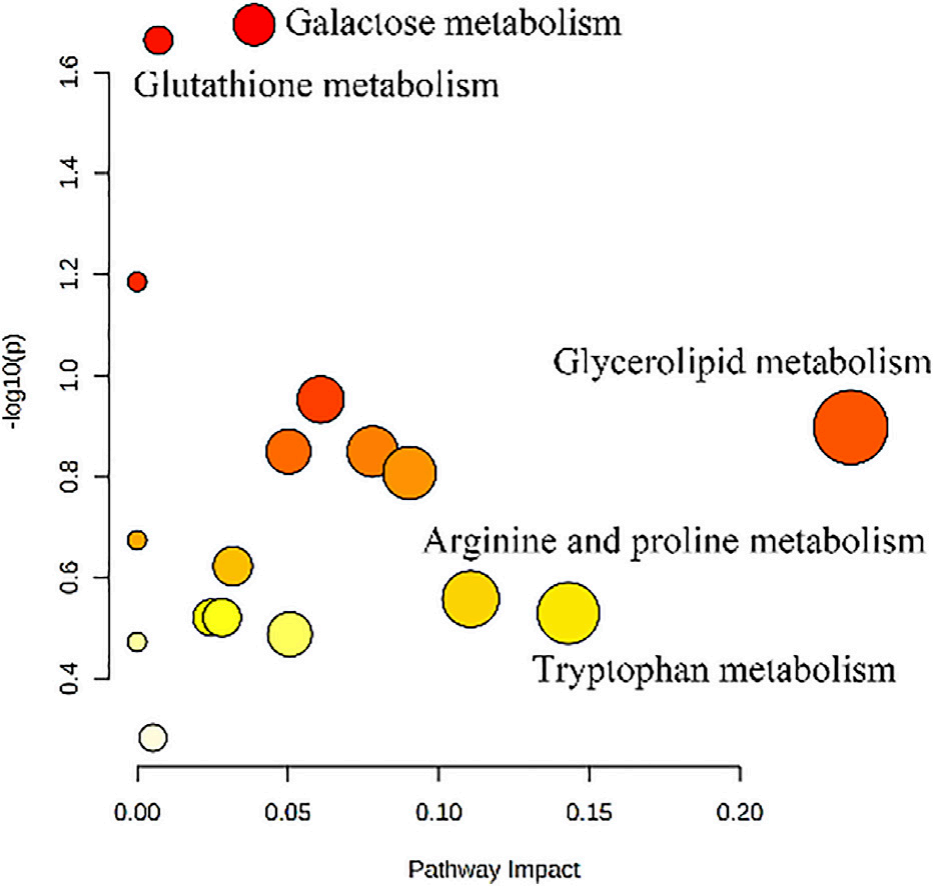
Pathways analysis of potential biomarkers.

The biomarkers in Table 1 were imported into MetaboAnalyst 5.0 (http://www.metaboanalyst.ca) for pathway analysis, and the metabolic pathways with *p* < 0.05 or Impact >0.1 were screened out as potential kidney-yang deficiency metabolic pathways. As shown in Figure 7, five related metabolic pathways were finally screened out, namely Galactose metabolism, Glutathione metabolism, Glycerolipid metabolism, Arginine and proline metabolism and Tryptophan metabolism.

Analysis of potential biomarkers of kidney-yang deficiency and related metabolic pathways suggested that the processing excipient of suet oil might play a role in warming kidney and promoting Yang by regulating Galactose metabolism. Epimedium raw product and Epimedium heating product might improve kidney-yang deficiency through Galactose metabolism, Glutathione metabolism and Arginine and proline metabolism. Meanwhile, the metabolic pathways above were thel metabolic pathways of Epimedium fried with suet oil to improve the kidney yang deficiency syndrome in rats, which further explained that the processing mechanism of epimedium could enhance its effect of warming kidney and promoting Yang after heating with the auxiliary material suet oil.

### 3.4 Network pharmacological analysis

**FIGURE 8:**
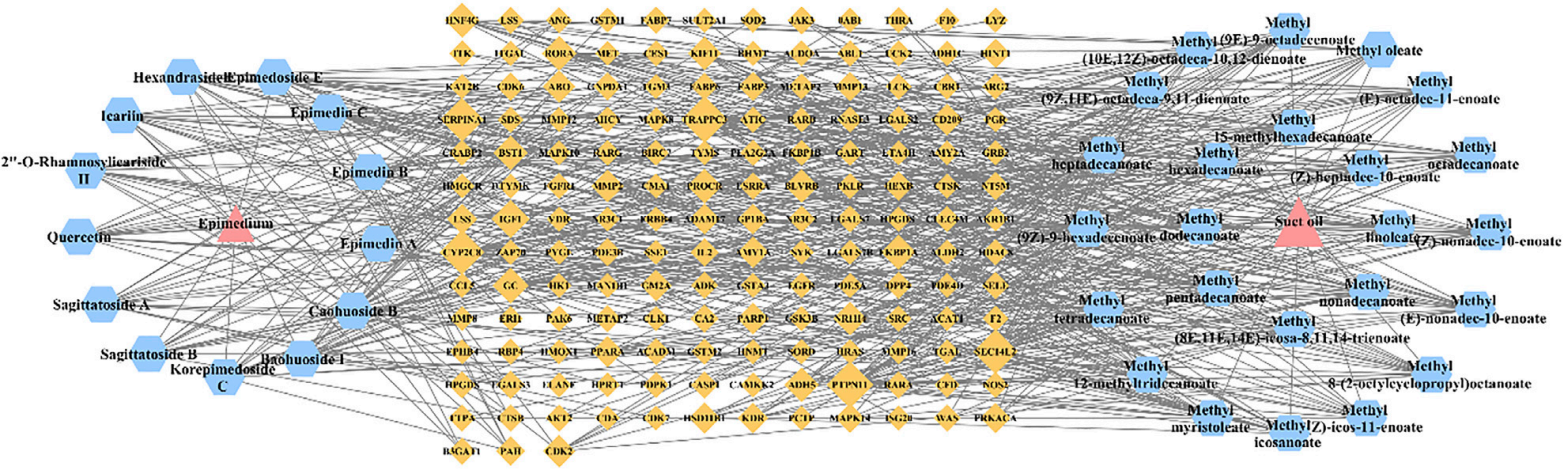
Pharmacology network of the “drug-active compounds-target” regulated by Epimedium fried with suet oil pink triangles indicate drugs, blue hexagons indicate active compounds, yellow diamonds indicate target genes.

Thirty-seven compounds were screened out as the active compounds library of Epimedium fried with suet oil, including 13 from Epimedium and 24 from suet oil. The specific results are listed in Supplementary Table S4. *Homo sapiens* species were selected to draw the protein-protein interaction (PPI) network of 159 common targets (Supplementary Figure S8). Then in order to reflect the relationship between the active compounds of Epimedium and suet oil and target genes in Epimedium fried with suet oil, a drug-active compounds-target network was constructed, as shown in the Figure 8. KEGG enrichment analysis was performed on 159 potential targets of active components of Epimedium fried with suet oil using Metascape database, and 267 pathways were obtained (Supplementary Figure S9).

### 3.5 Integrative analysis of the network pharmacology and metabolomics

**FIGURE 9:**
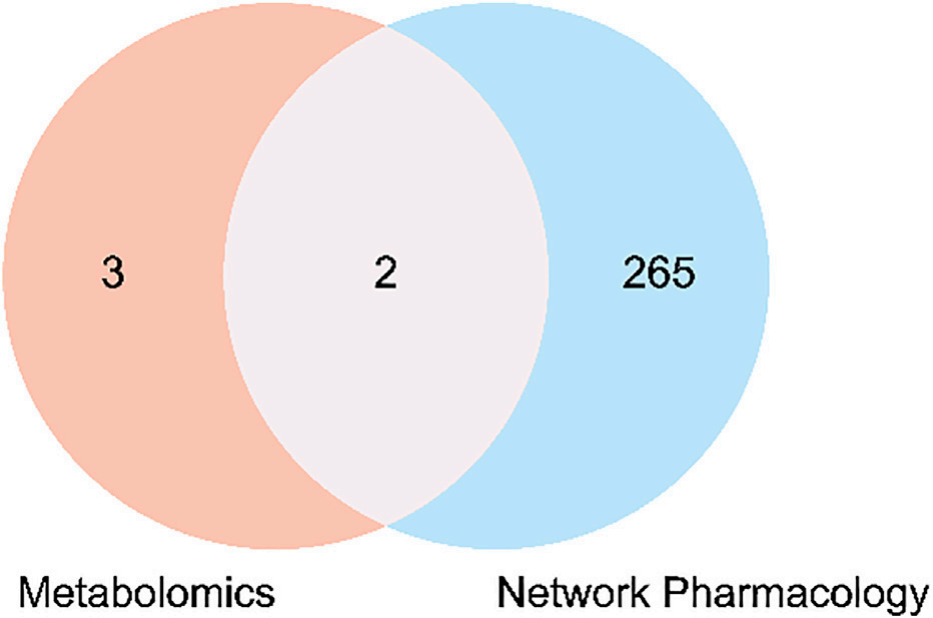
Overlapping pathways between metabolomics and network pharmacology analysis.

**FIGURE 10:**
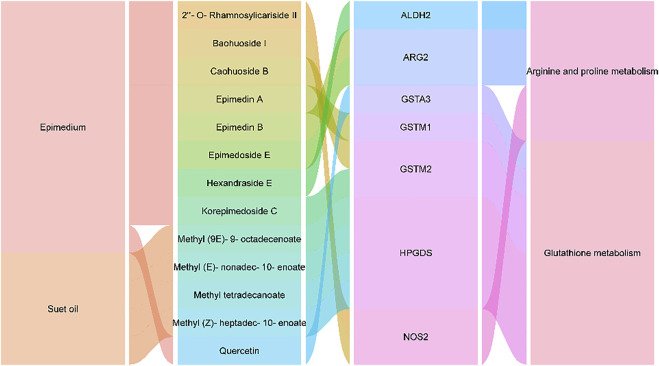
The network of drug-active compounds-target genes-pathways.

The five key metabolic pathways in metabolomics were mapped to the pathways of network pharmacology for analysis as shown in the Figure 9. It was found that two metabolic pathways (glutathione metabolism, arginine and proline metabolism) overlapped with it, and the corresponding 13 active compounds (9 active compounds in Epimedium and 4 in suet oil) and 7 target genes were obtained by reverse mapping analysis. Thirteen active compounds were Quercetin, Epimedin A, Caohuoside B, Korepimedoside C, Hexandraside E, Epimedin B, Epimedoside E, Baohuoside I, 2″-O-Rhamnosylicariside II, Methyl tetradecanoate, Methyl (Z)-heptadec-10-enoate, Methyl (9E)-9-octadecenoate, Methyl (E)-nonadec-10-enoate. The seven target genes were acetaldehyde dehydrogenase 2 (ALDH2), arginase type II (ARG2), glutathione S-transferase A3 (GSTA3), glutathione S-transferase M1 (GSTM1), glutathione S-transferase M2 (GSTM2), hematopoietic prostaglandin D synthase (HPGDS), Nitric Oxide Synthase 2 (NOS2). The drug-active compounds-target genes-pathways network was constructed as shown in Figure 10.

### 3.6 Experiment validation

**FIGURE 11:**
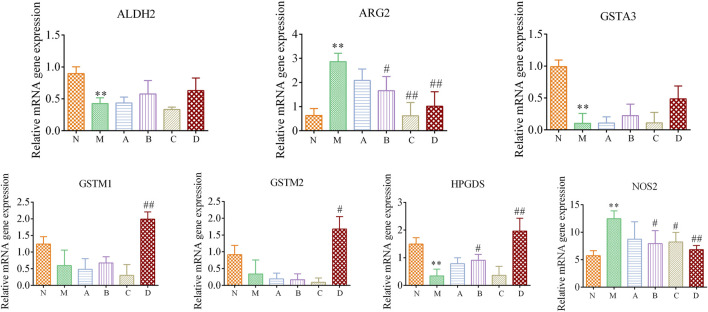
Epimedium fried with suet oil regulates differential gene expression associated with kidney yang deficiency. All genes expression were examined by RT-qPCR and normalized to GAPDH expression. Data represent mean ± SD for at least three independent experiments. Compared with the N group, *p < 0.05, **p < 0.01; compared with the M group ^#^ *p* < 0.05, ^##^ *p* < 0.01.

The RT-qPCR verification results were shown in Figure 11. Seven target genes were expressed in kidney tissue. Compared with the N group, the expression levels of two target genes (ARG2 and NOS2) in the M group were significantly increased (*p* < 0.01). The expression of five target genes was decreased, and which of three target genes (ALDH2, GSTA3, HPGDS) were significantly decreased (*p* < 0.01). Compared with the M group, the A, B, C and D groups had different degrees of callback effect on seven target genes. ALDH2, ARG2, GSTA3, HPGDS, NOS2 had a callback trend in the A group, but there was no significant difference. The expression of HPGDS target genes was significantly increased in the B group (*p* < 0.05). The expression of ARG2 and NOS2 were significantly decreased in the B and C groups (*p* < 0.05). The expression levels of GSTM1, GSTM2 and HPGDS were significantly increased (*p* < 0.05, *p* < 0.01), ARG2 and NOS2 expressions were significantly decreased (*p* < 0.01) in the D group.

The callback results of the target genes in each administration group showed that the improvement effect of Epimedium fried with suet oil on kidney-yang deficiency was better than that of the other three administration groups. Notably, the five target genes with significant callback effects in Epimedium fried with suet oil were derived from the target genes corresponding to the active compounds of Epimedium (GSTM1, GSTM2 ARG2 and NOS2) and the target gene corresponding to the active compounds of suet oil (HPGDS). Furthermore, the synergistic mechanism of Epimedium fried with suet oil to warm the kidney and enhance yang was clarified.

### 3.7 Overall interactive network diagram of drugs, active compounds, targets, pathways, and metabolites

**FIGURE 12:**
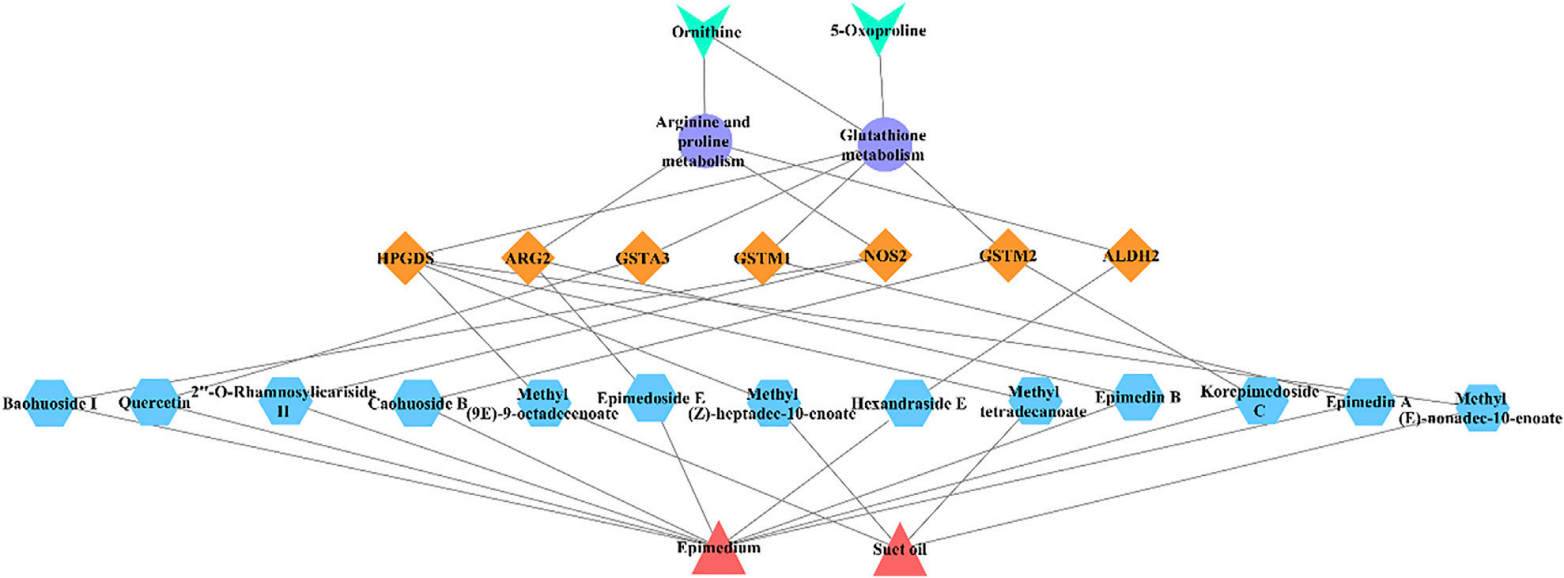
The overall interactive network diagram pink triangles indicate drugs, blue hexagons indicate active compounds, yellow diamonds indicate target genes, purple ellipses indicate pathways, green “V” indicates biomarks.

Figure 12 showed the overall interaction network diagram of drugs, active compounds, targets, pathways and metabolites. Integrating network pharmacology, metabolomics and RT-qPCR analysis, it was found that Epimedium fried with suet oil mainly affects 13 active compounds, seven targets, and two biomarkers in two metabolic pathways, thereby improving kidney-yang deficiency. Its mechanism of action may mainly involve amino acid metabolism and oxidative stress.

## 4 Discussion

Epimedium is a TCM for kidney-yang deficiency. The most common processing method is suet oil processing, which aims to enhance the kidney-warming and yang-boosting effects of Epimedium. Previous studies have shown that the model of kidney yang deficiency was established through glucocorticoid, and its mechanism was to simulate the pathological state when the hypothalamic-pituitary-target gland axis (adrenal gland, thyroid gland, gonad) was inhibited, thereby producing deficiency symptoms similar to “kidney yang deficiency” (Zhang et al., 2014). Kidney-yang deficiency is manifested in macroscopic characterization (e.g., weight loss), biochemical standards (e.g., decreased hormone levels of CORT, 17-OHCS, T3, T4, and T), and organ histopathology (e.g., pathological changes in the hypothalamus, pituitary gland, thyroid gland, adrenal gland, and testis). This study found that compared with the N group, the body weight of the M group rats was reduced, and the levels of hypothalamic-pituitary-adrenal axis hormones (CORT, ACTH, 17-OHCS), hypothalamic-pituitary-thyroid axis hormones (T3, T4, TSH) and hypothalamic-pituitary-gonadal axis hormones (LH, T, FSH) were inhibited. Pathological changes occurred in the tissues and cellular structures of hypothalamus, pituitary gland, thyroid gland, adrenal gland, testis and kidney. Compared with M group, in addition to the A group, B, C, and D groups could reverse this inhibition of different level and improve the pathological changes of the organization, and the reverse effect of Epimedium fried with suet oil was better than that of raw epimedium and heated epimedium, suggesting that the effect of warming the kidney and promoting yang of epimedium is enhanced after being heated and processed with suet oil.

Subsequently, in this study, we analyzed the processing synergistic mechanism of Epimedium fried with suet oil through metabonomics combined with network pharmacology. In the metabolomic results, 15 biomarkers related to kidney-yang deficiency were screened in urine and plasma, involving five metabolic pathways, namely Galactose metabolism, Glutathione metabolism, Glycerolipid metabolism, Arginine and proline metabolism, Tryptophan metabolism. Among them, Galactose metabolism was related to energy metabolism, while Glutathione metabolism, Glycerolipid metabolism, Arginine and proline metabolism, and Tryptophan metabolism were closely related to amino acid metabolism and oxidative stress. Galactose metabolism regulated by suet oil and Galactose metabolism, Glutathione metabolism, Arginine and proline metabolism regulated by Epimedium heating product were the metabolic pathways of Epimedium fried with suet oil group to improve kidney yang deficiency in rats. In the network pharmacology results, seven target genes (ALDH2, ARG2, GSTA3, HPGDS, GSTM1, GSTM2, and NOS2) and two linked metabolic pathways (glutathione metabolism, arginine and proline metabolism) were identified by the drug-active compounds-target genes-pathways network.

Glutathione comprises cysteine, glutamic acid and glycine. The main metabolic pathways of glutathione include glutathione producing glutathione disulfide (GSSG) under the action of glutathione peroxidase (GSHPx) and cross-linking complex under the action of glutathione transferase (GST). It plays a role in oxidative stress, participating in cell apoptosis and regulating signal transduction (Xu et al., 2014). Studies have found that genes related to kidney-yang deficiency in the aged are closely related to aging (Dong et al., 2013), and glutathione deficiency can induce oxidative stress in the aging process. Increasing the uptake of the glutathione, precursors glycine and cysteine, can restore the synthesis and concentration of glutathione and reduce oxidative stress and oxidative damage in the aging process (Sekhar et al., 2011). (Du et al., 2020) used LC-MS metabolomics method to identify 31 biomarkers related to kidney Yang deficiency from testicular tissue, and 26 of them could be significantly recovered after Gulingji intervention. It was speculated that they might improve kidney Yang deficiency syndrome by regulating glutathione metabolism, arginine and proline metabolism, glyoxylate and dicarboxylic acid metabolism and other pathways. In this study, it was found that Ornithine and 5-Oxoproline are both products of glutathione metabolism and biomarkers of kidney Yang deficiency. Epimedium fried with suet oil could play its role in warming kidney and helping Yang by regulating these two biomarkers of kidney Yang deficiency. GSTA3, GSTM1 and GSTM2 are transferases of glutathione metabolism, which can catalyze the cross-linking complex between glutathione and electron-philic complex to protect cells from free radical damage. HPGDS, also known as glutathione dependent prostaglandin D synthetase, is involved in the biosynthesis of prostaglandin in adult testis, thereby affecting the production of spermatogenesis and the normal function of testis (Körber and Goericke-Pesh, 2019). The active components of Epimedium fried with suet oil, Quercetin, Epimedin A, Caohuoside B and Korepimedoside C, mainly acted on glutathione sulfur transferases (GSTA3, GSTM1 and GSTM2). Methyl tetradecanoate, Methyl (Z)-heptadec-10-enoate, Methyl (9E)-9-octadecenoate and Methyl (E)-nonadec-10-enoate mainly acted on HPGDS, and then affected glutathione metabolism.

Arginine and proline metabolism is the core process of the biosynthesis and metabolism of arginine, ornithine, proline, citrulline and glutamate (Feng et al., 2022). Arginine is a semi-essential amino acid synthesized from glutamine, glutamate, and proline *via* the gut-kidney axis in humans and most mammals, and plays an important role in the human body as a precursor of various physiologically important substances (Ma et al., 2019). Several studies have shown that arginine and its derivatives may be involved in the pathogenesis of kidney diseases and are markers of pathological progression (Feng et al., 2020). Arginine is the precursor of the synthesis of ornithine, which participates in the ornithine cycle under the action of ornithine carbamoyl transferase, promotes the synthesis of urea and accelerates the degradation of blood ammonia. Arginine can form ornithine and urea under the action of arginase, and produce nitric oxide under the action of nitric oxide synthase (Castilho-Martins et al., 2015). These pathways are involved in biochemical processes such as ammonia detoxification, hormone secretion and immune system regulation (Chang et al., 2020). The level of ornithine increased in kidney-yang deficiency rats. It was speculated that the regulation of ornithine in Epimedium fried with suet oil group might be related to the adjustment of gut microbiota balance, the reduction of renal tubulointerstitial fibrosis and the improvement of renal function. In this study, Hexandraside E, the active component of Epimedium fried with suet oil, mainly acted on ALDH2. Epimedin B and Epimedoside E mainly acted on ARG2. Baohuoside I and 2″-O-Rhamnosylicariside II mainly acted on NOS2. Thus, they jointly regulated arginine and proline metabolism.

Galactose metabolism is closely related to glycolytic metabolic pathways, which can reflect the level of energy metabolism of the body and is important for stabilizing the structure of the whole branched carbohydrate chain (Guo et al., 2016; Jin et al., 2019). Galactose metabolism is not only important for energy production in cells, but also contributes to the modification of glycolipids and glycoproteins (Petry and Reichardt, 1998). Galactose metabolism may be associated with chronic inflammation. When galactose metabolism is disrupted, it also promotes the release some inflammatory factors to participate in the body’s inflammatory response (Nikolac Perkovic et al., 2014). It is found that the liver and kidney of KYDS rats may be damaged due to the effect of hydrocortisone, thus affecting galactose metabolism (Chen et al., 2018). In this paper, the rats with kidney yang deficiency have cold phobia, kidney damage and other deficiency symptoms, indicating that their energy metabolism is abnormal. After administration, the abnormal galactose metabolism in the body gradually returned to normal, the body’s energy metabolism also tended to return to normal, and the inflammation was improved.

Glycerolipid metabolism is one of the important ways of lipid metabolism in the body, and the core reaction is the acetylation of glycerol and the degradation of triacylglycerol (Li et al., 2022). Lipids are divided into eight groups, including fatty acids, triglycerides, glycerolipids, sphingolipids, glycolipids, polyketones, sterolipids, and isopentenol lipids (Fahy et al., 2005). Changes in lipid metabolites can lead to a series of pathophysiological phenomena, including obesity (Nam et al., 2018), inflammation etc, (Ralston et al., 2017). Disturbance of Glycerolipid metabolism was observed in the early, middle and late stages of Aristolochic acid nephropathy (Zhao et al., 2015). Glycerol, as an important small molecule, participates in the Glycerolipid metabolism (Liu et al., 2019). The results of this experiment showed that glycerol, a differential marker screened, had an effect on Glycerolipid metabolism. Compared with the Control group, glycerol content in the kidney-yang deficiency model group was significantly decreased. Different administration groups could adjust the contents to different degrees, especially the callback effect of the D group had the most significant.

Tryptophan, as an essential amino acid, is involved in tryptophan metabolism (Liu et al., 2022). Tryptophan metabolism is closely related to the central nervous system because tryptophan is involved in serotonin synthesis (Kałużna-Czaplińska et al., 2019). Tryptophan metabolism is related to the activity of inflammatory bowel disease and has significant effects on gastrointestinal physiology (Hua et al., 2019). Zhou et al. found that tryptophan metabolism was seriously disturbed in KYDS state in the study of the therapeutic effect of ShenQiWan on kidney yang deficiency (Zhou et al., 2016). Tong et al. found that tryptophan is a precursor of serotonin, which is closely related to KYDS metabolic disorders requiring the participation of intestinal flora (Tong et al., 2022). In this study, tryptophan metabolism was disturbed in the model group of kidney-yang deficiency rats. This is consistent with the results of the literature. Compared with the control group, the tryptophan level in the model group decreased significantly. Epimedium fried with suet oil could improve the disorder of tryptophan metabolism by significantly regulating the tryptophan metabolite, showing obvious anti KYDS effect.

To further verify the results, RT-qPCR was used to analyze the expression levels of the seven selected targets in the kidney tissues of the six groups of rats. GSTA3, GSTM1, GSTM2, and HPGDS belong to the glutathione sulfur transferase series, which are related to prostaglandin synthesis and participates in glutathione metabolism. GSTA3, GSTM1, and GSTM2 are widely present in various tissues and cells of the body, and are related to the occurrence of cell damage, oxidative stress, poisoning, aging and other disease processes (Chen et al., 2018). Glutathione thitransferase A3 (GSTA3), as one of the most important members of the glutathione transferase family, is involved in detoxication and cell protection (Ilic et al., 2010). GSTA3 has a protective effect on Tubular epithelial-mesenchymal transition in renal fibrosis (Xiao et al., 2016). Glutathione S transferase M1 (GSTM1) is an important member of phase II toxic metabolizing enzyme GST family, which plays a detoxification role by binding with glutathione (Mehdi et al., 2019). It is reported that GSTM1 can repair acute kidney injury by reducing endoplasmic reticulum and oxidative stress (Dai et al., 2021). Glutathione S-transferase M2 (GSTM2) is a protein involved in the detoxification of reactive oxygen species (Zhou et al., 2008). GSTM2 has the activity of prostaglandin E synthetase (PGES) and can participate in the synthesis of prostaglandin E2 (PGE2) (Beuckmann et al., 2000).

HPGDS is a member of the glutathione S-transferases (GSTs) family that is closely associated with inflammatory diseases (Huang et al., 2021). HPGDS is mainly expressed in hematopoietic cell lines, catalyzes the synthesis of prostaglandin D2, and reduces inflammation by regulating the apoptosis of T cells and B cells (Rittchen and Heinemann, 2019). It was found that icariin can increase the levels of glutathione sulfotransferase and glutathione, and decrease the levels of malondialdehyde and nitric oxide (Amanat et al., 2022). In this study, the expression levels of GSTA3, GSTM1, GSTM2 and HPDGS were decreased in kidney tissues after modeling, suggesting that kidney Yang deficiency was accompanied by apoptosis, oxidative stress injury and inflammation of kidney tissue cells, and then caused kidney injury. This is consistent with the kidney histopathological results described above. However, D group could reverse the above level expression. Therefore, it could be speculated that Epimedium fried with suet oil might alleviate kidney injury caused by apoptosis of kidney tissue by increasing the level of glutathione transferase, and then played the role of warming kidney and helping Yang.

ALDH2 is related to the body’s energy metabolism and carbohydrate metabolism (Guo et al., 2020). ALDH2 is an important endogenous cardioprotective factor in mitochondria (Xia et al., 2023). Studies have shown that acetaldehyde dehydrogenase ALDH2, as a key enzyme in alcohol metabolism in human body, may affect the occurrence and development of coronary heart disease by affecting the level of blood glucose metabolism (You et al., 2018). ALDH2 plays an important regulatory role in the occurrence and development of tumors, and has a key role in maintaining tumor cell stemness (Toledo-Guzmán et al., 2019). ALDH2 is related to glycolysis and affects the level of blood glucose metabolism, which is consistent with the results of kidney yang deficiency involving Galactose metabolism pathway in this study. In this study, it was found that the expression level of ALDH2 in the kidney tissue of the M group was significantly decreased. It was speculated that the expression level of these related target genes was decreased during the occurrence of kidney Yang deficiency, resulting in the inhibition of the body’s energy metabolism, which was consistent with the literature that kidney Yang deficiency caused the body’s low energy metabolism (Chen et al., 2018). After administration, the callback trend of D group was closest to that of the N group. This suggested that Epimedium fried with suet oil could promote the expression of ALDH2, improve the body’s energy metabolism, and then improve the state of kidney Yang deficiency.

ARG2 is mainly expressed in kidney, brain and prostate, and located in mitochondria. In clinical studies, arginase activation is closely related to the occurrence and development of heart, lung and kidney ischemia-reperfusion injury, hypertension, erectile dysfunction, atherosclerosis, aging and other diseases (Wu et al., 2015). Studies have shown that cell aging caused by aging is associated with increased intracellular ARG2 activity/expression (Meng and Xiong, 2016). Inhibition of arginase I leads to increased expression of NOS2, thus promoting the production of NO (Raber et al., 2012). In this study, it was found that ARG2 expression level was significantly increased in the M group, suggesting that kidney Yang deficiency might be related to the increased expression level of ARG2 in the kidney tissue, the decreased content of NO and the increased level of superoxide production, which may lead to renal endothelial dysfunction. However, Epimedium fried with suet oil could significantly reduce the ARG2 expression levels, indicating that it might play its role by reducing ARG2 expression levels and alleviating the endothelial dysfunction caused by its overexpression.

NOS2 is the rate-limiting enzyme of NO synthesis, which plays its biological role by producing NO and has important clinical significance in the occurrence and development of diseases (Mount and Power, 2006). Excessive production of NO by NOS2 will lead to cell damage and tissue necrosis, and further promote the occurrence and development of inflammatory diseases. NOS2 is an important intracellular messenger and molecular marker in the mechanism of inflammation (Jiang et al., 2016). In the kidney, NO is involved in the regulation of renal vascular resistance, glomerular filtration rate and maintenance of renal structural integrity (Da Silva et al., 2021). Many studies have shown that NOS2 plays a key role in the pathogenesis of metabolic nephropathy, and it has been found in the pathogenesis of chronic nephropathy that elevated NOS2 levels produce highly reactive NO, which is associated with chronic low-grade inflammatory states (Zhang et al., 2021). In this study, the expression level of NOS2 in the D group was significantly decreased. It suggested that Epimedium fried with suet oil could reduce the production of NO, reduce the inflammatory state of kidney tissue and maintain the integrity of kidney structure by reducing the overexpression of NOS2 in the kidney tissue.

## 5 Conclusion

In this study, 15 differential metabolites in urine and plasma were screened by GC-MS technique. The regulatory effect of Epimedium on abnormal metabolism of kidney Yang deficiency was related with Galactose metabolism, Glutathione metabolism, Glycerolipid metabolism, Arginine and proline metabolism, Tryptophan metabolism. The processing excipient “suet oil” regulated Galactose metabolism, and “heating” regulated Galactose metabolism, Glutathione metabolism, Arginine and proline metabolism. Together, the two factors enhanced the role of Epimedium fried with suet oil in warming kidney and enriching yang. This further explained the processing mechanism of synergistic effect of Epimedium processed by heating and suet oil. Based on the results of metabolomics and network pharmacology, combined with RT-PCR verification, the results showed that the 13 active components of Epimedium fried with suet oil played the role of warming kidney and promoting Yang by acting on the seven target genes, two pathways and two biomarkers of kidney Yang deficiency, which further elucidated the processing and synergistic mechanism of Epimedium fried with suet oil. In view of the characteristics of multi-component, multi-target and overall synergy of TCM, this study comprehensively characterized the mechanism of processing effect of Epimedium, which would help to systematically and profoundly reveal the processing mechanism of Epimedium fried with suet oil. At the same time, it provides a research strategy for studying the processing mechanism of TCM and the action mechanism of TCM in treating deficiency syndrome.

## Data Availability

The original contributions presented in the study are included in the article/supplementary material, further inquiries can be directed to the corresponding authors.
