# Supplementary material for: Integrating strategies of metabolomics, network pharmacology, and experiment validation to investigate the processing mechanism of Epimedium fried with suet oil to warm kidney and enhance yang
Source: Front Pharmacol. 2023 Jan 25;14:1113213. doi: 10.3389/fphar.2023.1113213 (PMC9905240; doi:10.3389/fphar.2023.1113213)
Supplement: Supplementary file 1 [file DataSheet1.docx]

Supplementary Material

Integrating Strategies of Metabolomics, Network Pharmacology, and Experiment Validation to Investigate the Processing Mechanism of Epimedium Fried with Suet Oil to Warm Kidney and Enhance Yang

E Sun^1,2†*^, Ran Huang^1,3†^, Ke Ding^1^, Ling Wang^1^, Jian Hou^2^, Xiaobin Tan^1,2^, Yingjie Wei^1,2^, Liang Feng^4^, Xiaobin Jia^1,4*^

^1^The Third Clinical Medical College, Nanjing University of Chinese Medicine, Nanjing 210028, China

^2^Key Laboratory of New Drug Delivery System of Chinese Materia Medica, Jiangsu Academy of Traditional Chinese Medicine, Nanjing 210028, China

^3^Affiliated Hospital of Nanjing University of Chinese Medicine, Nanjing 210029, China

^4^School of Traditional Chinese Pharmacy, China Pharmaceutical University, Nanjing 211198, China

^†^These authors have contributed equally to this work and share first authorship

*** Correspondence:** E Sun, [sune0825@163.com](mailto:sune0825@163.com); Xiaobin Jia, jiaxiaobin2015@163.com

# Supplementary Figures and Tables

## Supplementary Figures


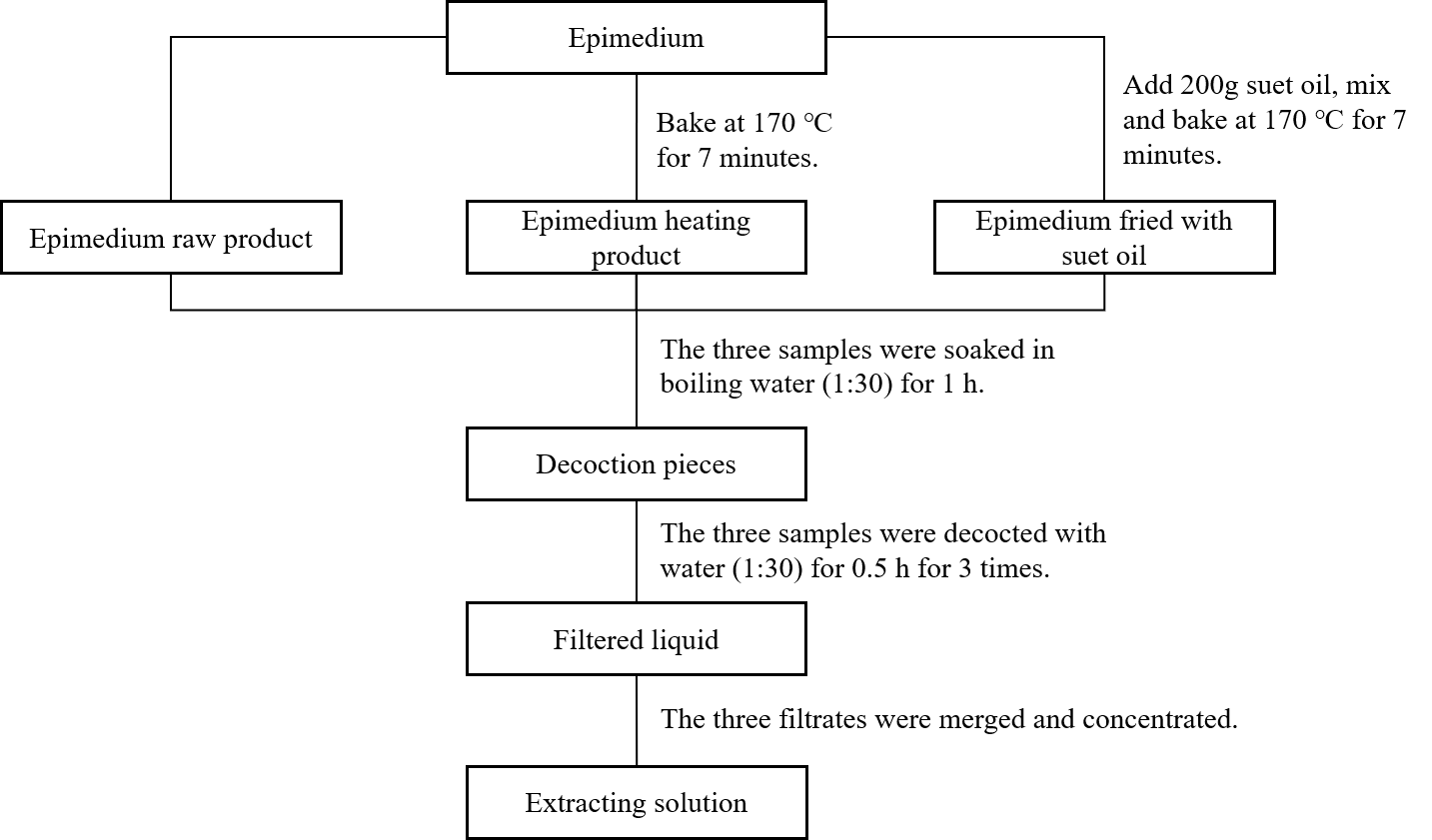


**Supplementary Figure 1.** Preparation of Epimedium processing and extraction


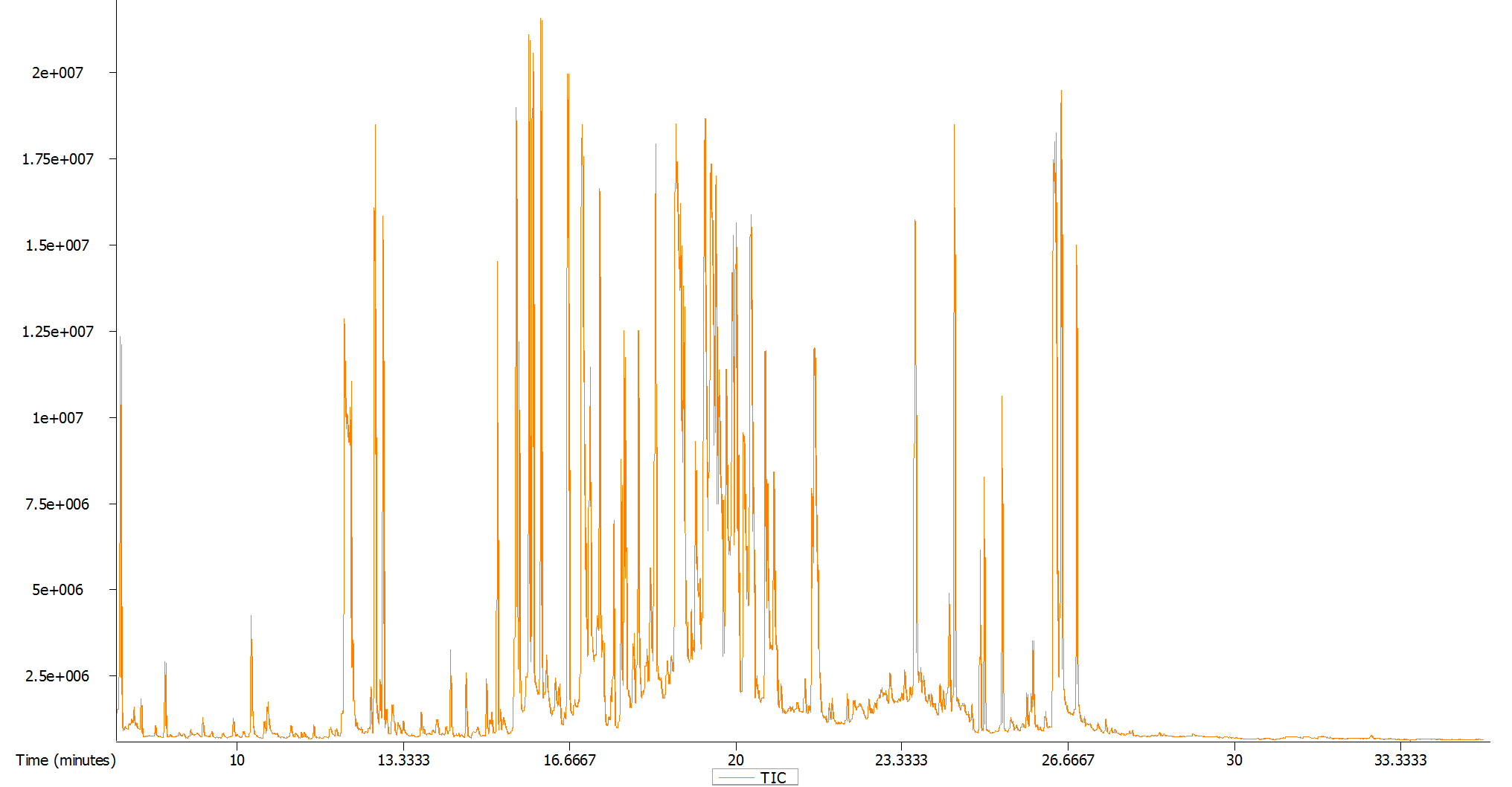


**Supplementary Figure 2.** GC-MS TIC of N2 urine sample


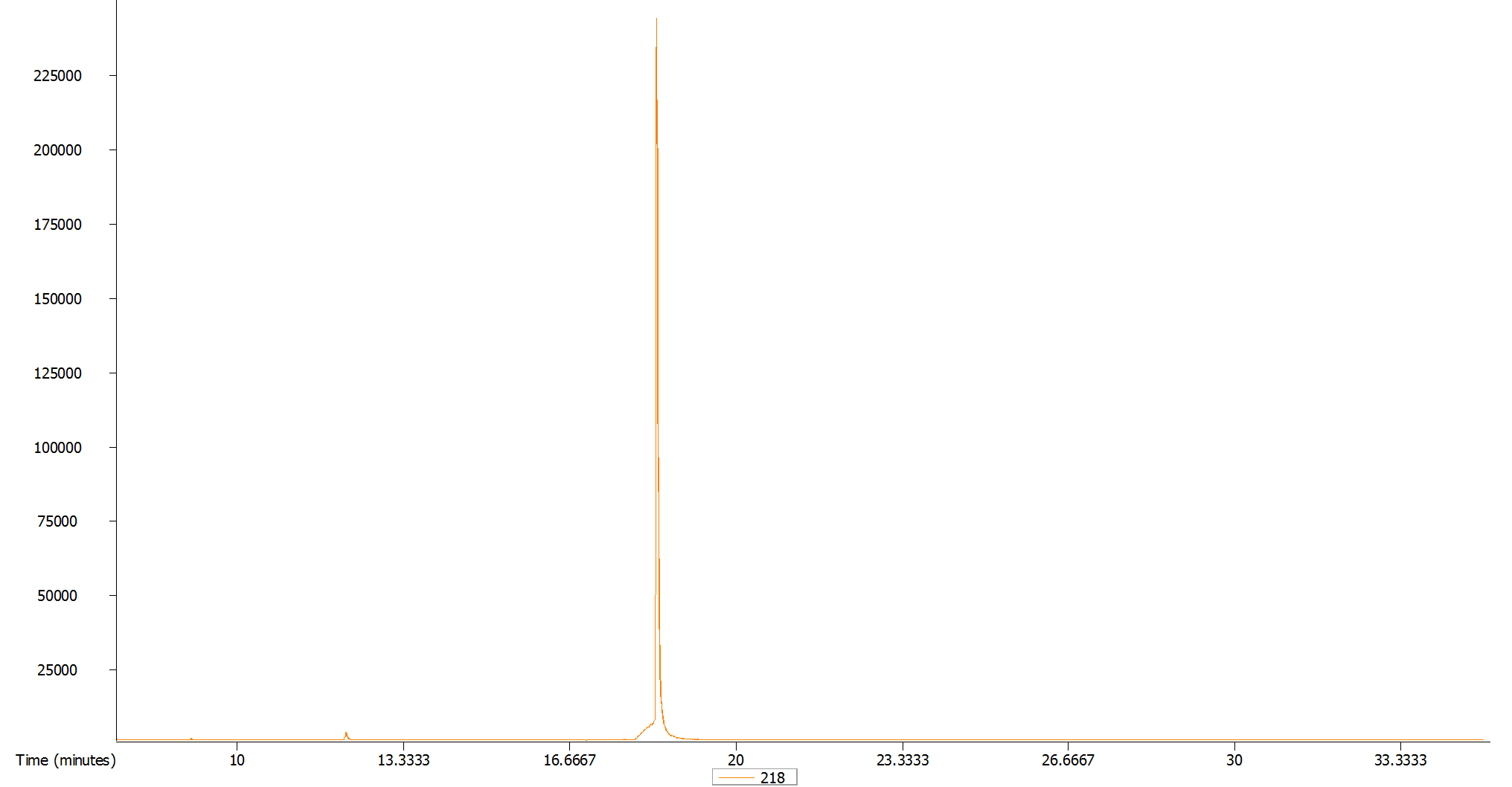


**Supplementary Figure 3.** GC-MS TIC of Internal standard (L-2-chlorophenylalanine)


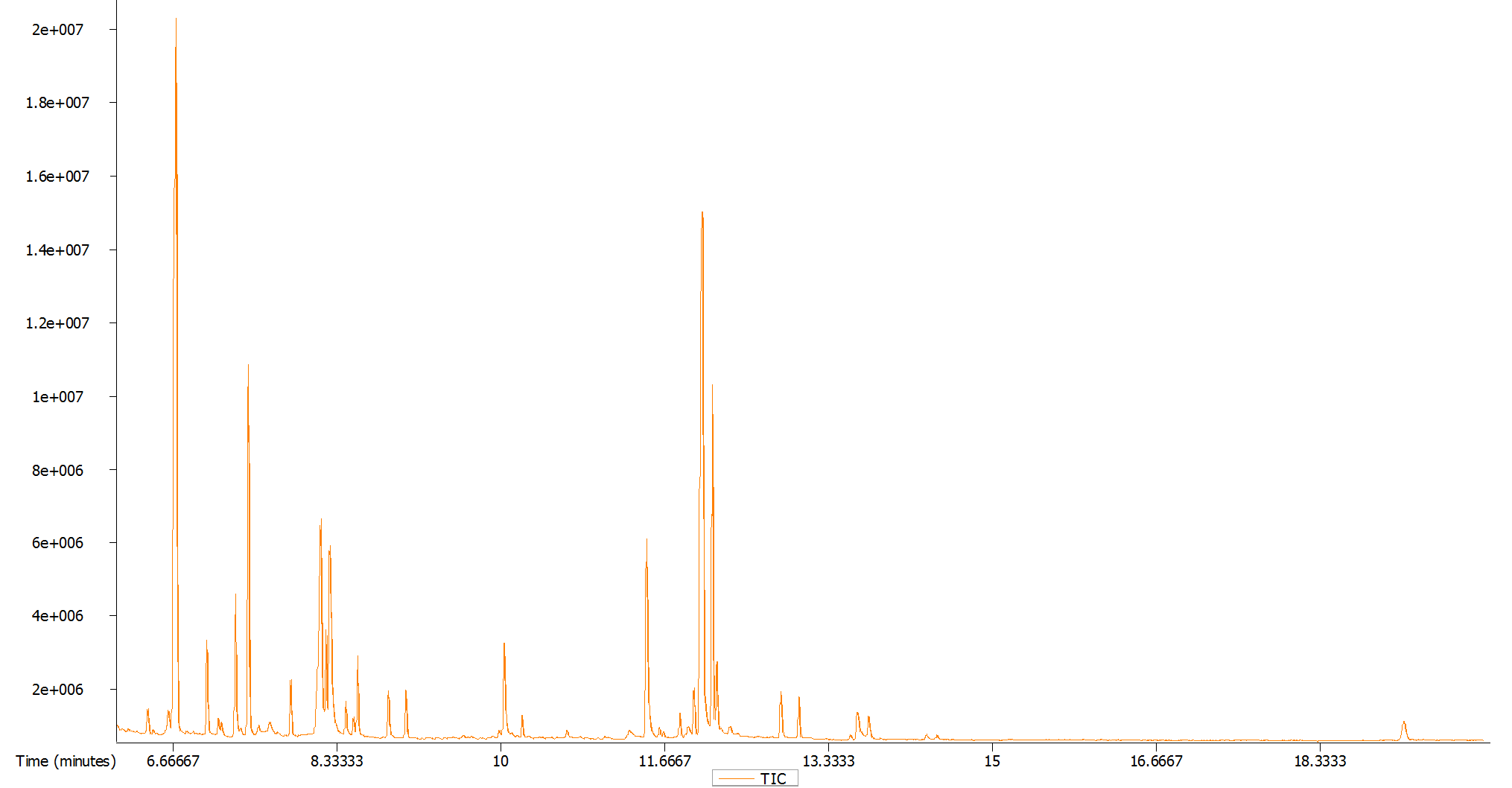


**Supplementary Figure 4.** GC-MS TIC of N5 plasma sample


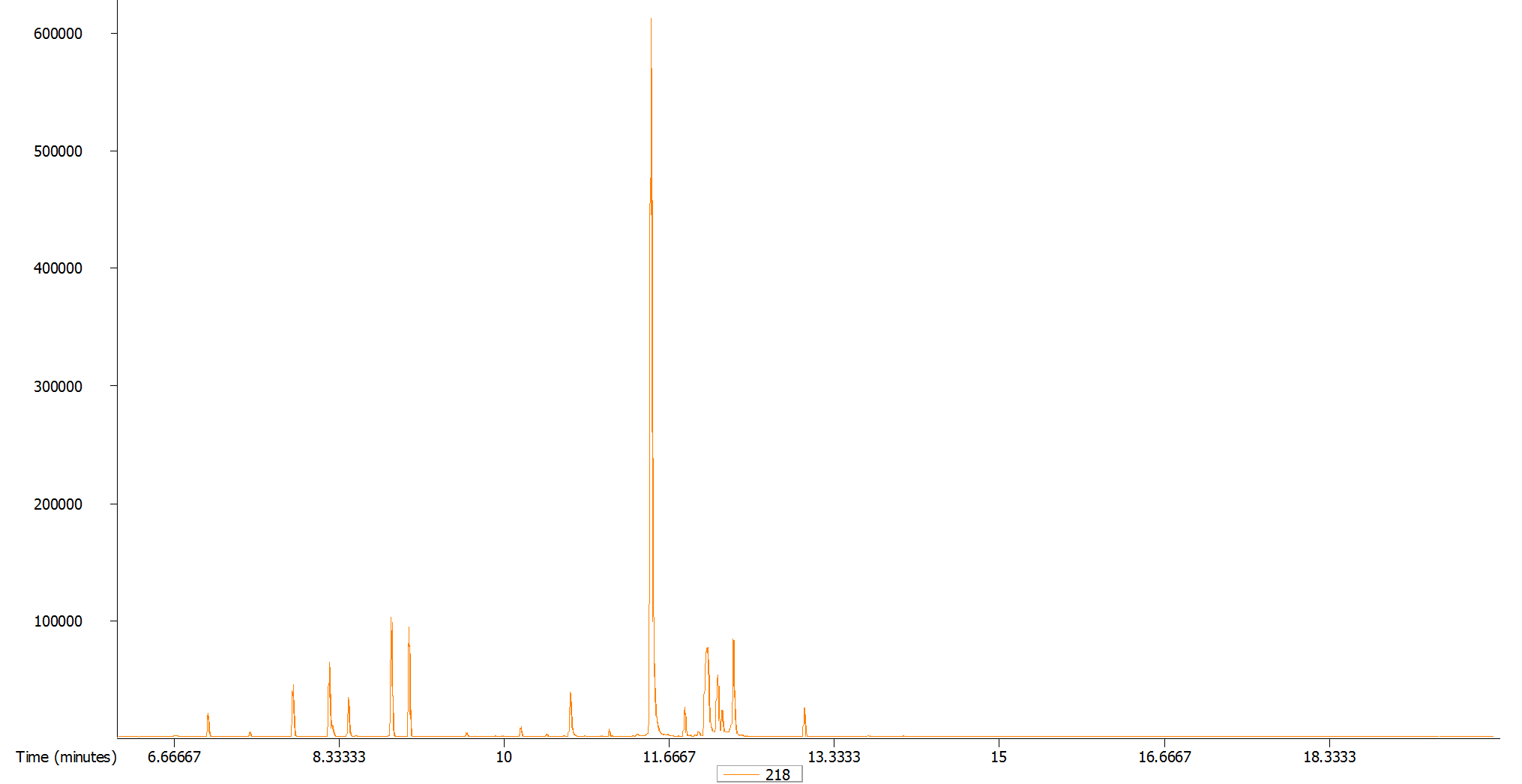


**Supplementary Figure 5.** GC-MS TIC of Internal standard (L-2-chlorophenylalanine)


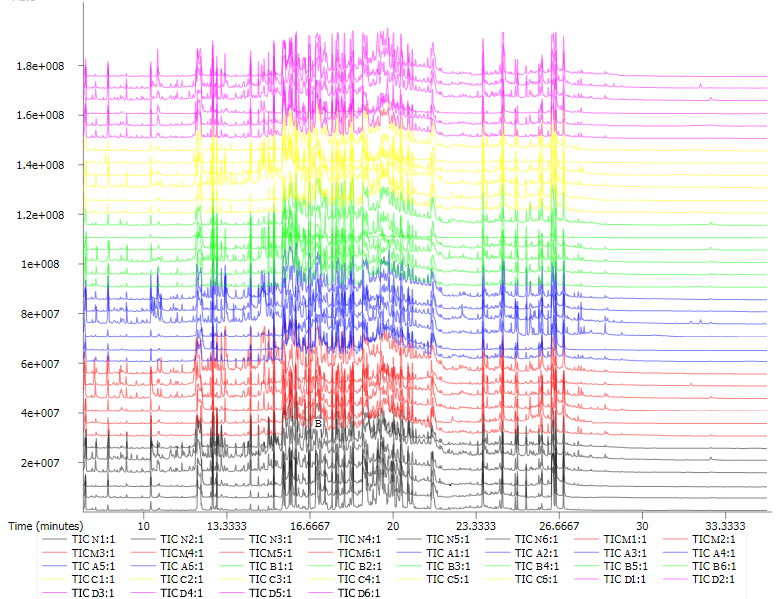


**Supplementary Figure 6.** GC-MS TIC of urine samples


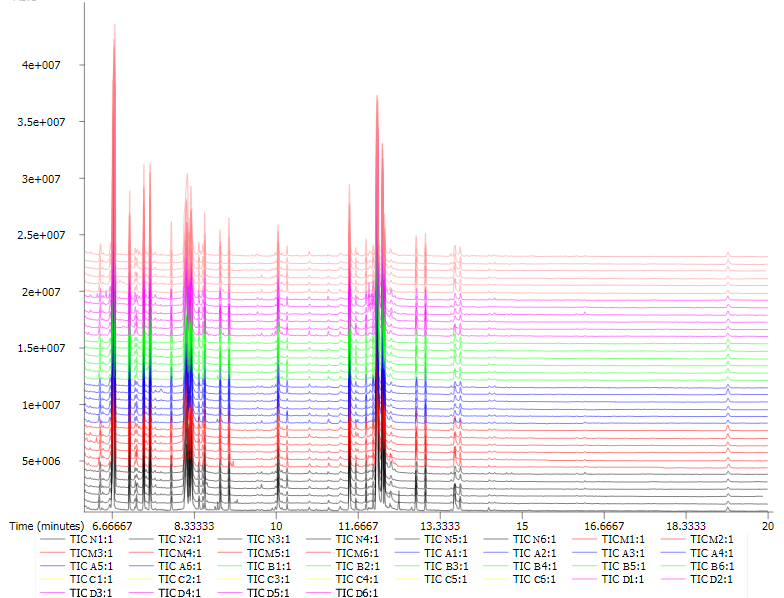


**Supplementary Figure 7.** GC-MS TIC of plasma samples


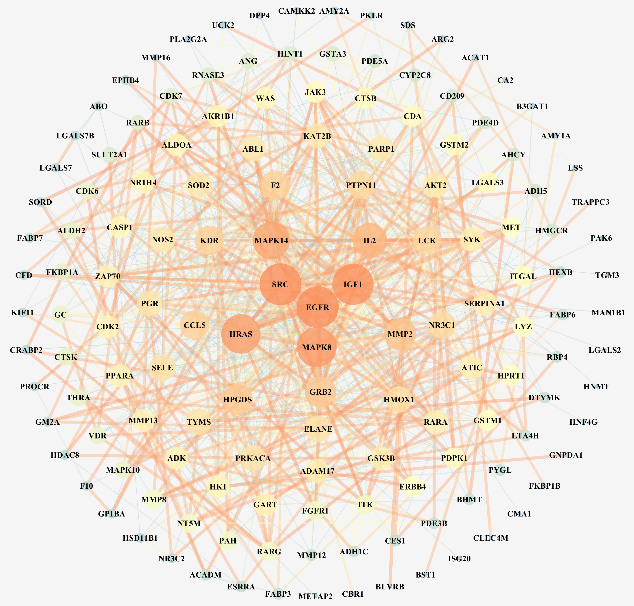


**Supplementary Figure 8.** Target genes identified in the protein-protein interaction network.


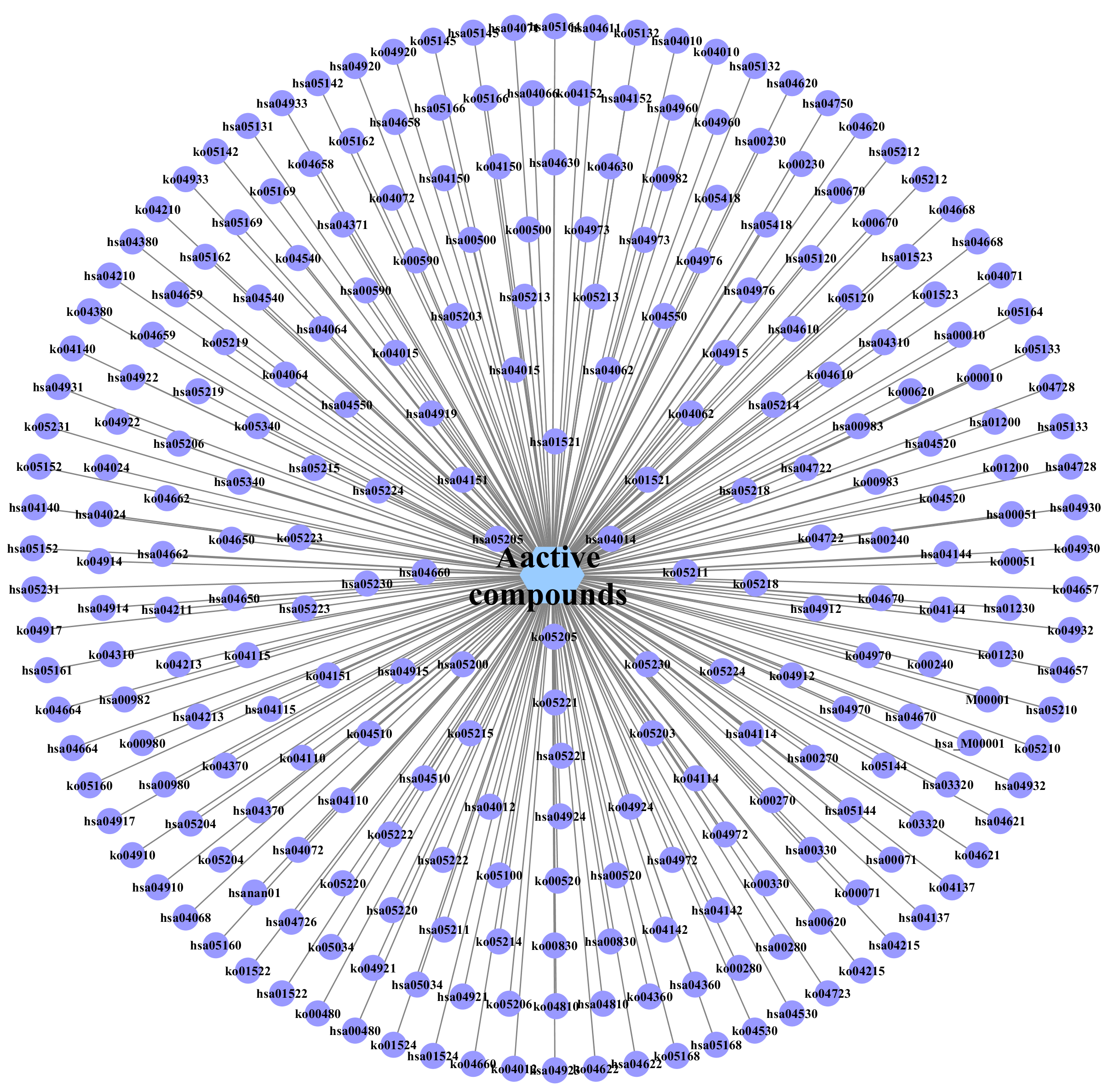


**Supplementary Figure 9.** Active compounds-pathways network (blue hexagon indicates 37 active compounds, purple ellipses indicate 267 pathways)

## Supplementary Tables

**Supplementary Table 1.** Primer sequence for quantitative real-time PCR analysis

| Gene | Primer Sequence (5'-3') | Length (bp) |
| --- | --- | --- |
| Aldehyde dehydrogenase, mitochondrial (ALDH2) | Forward Primer: AAGGGAACAAGGAGGACGTAGAC | 122 |
|  | Reverse Primer: ATCAGCCAATCGGTACAACAGC |  |
| Arginase-2, mitochondrial (ARG2) | Forward Primer: CATTGGCTTACGAGATGTGGAG | 141 |
|  | Reverse Primer: CTCTTGCCAATCAGCCGATC |  |
| Glutathione S-transferase A3 (GSTA3) | Forward Primer: TATGGGAAGGACATGAAGGAGA | 148 |
|  | Reverse Primer: GGTTCCTTGCTTTGTCCTTGA |  |
| Glutathione S-transferase Mu 1 (GSTM1) | Forward Primer: TGCAGCTCATCATGCTTTGTTA | 83 |
|  | Reverse Primer: CTTCATCTTCTCAGGGATGGTCT |  |
| Glutathione S-transferase Mu 2 (GSTM2) | Forward Primer: CTATGGACACCCGCCTACAGT | 257 |
|  | Reverse Primer: CTCAAACCGAGCCACGAAGT |  |
| Glutathione-requiring prostaglandin D synthase (HPGDS) | Forward Primer: ACTTCACCAGAGCCTCGCAATA | 113 |
|  | Reverse Primer: AATCGTCCAGGGTATCCACCA |  |
| Nitric oxide synthase, inducible (NOS2) | Forward Primer: CAGATCCCGAAACGCTACACTT | 175 |
|  | Reverse Primer: TGCGGCTGGACTTCTCACTC |  |
| glyceraldehyde-3-phosphate dehydrogenase (GAPDH) | Forward Primer: CTGGAGAAACCTGCCAAGTATG | 138 |
|  | Reverse Primer: GGTGGAAGAATGGGAGTTGCT |  |

**Supplementary Table 2.** Internal standard (L-2-chlorophenylalanine) retention time(urine sample)

| Sample | R.T. (min) | Sample | R.T. (min) |
| --- | --- | --- | --- |
| N1 | 18.3942 | B1 | 18.3958 |
| N2 | 18.3892 | B2 | 18.3942 |
| N3 | 18.3950 | B3 | 18.3992 |
| N4 | 18.3958 | B4 | 18.4000 |
| N5 | 18.4108 | B5 | 18.3892 |
| N6 | 18.3975 | B6 | 18.4033 |
| M1 | 18.3900 | C1 | 18.4025 |
| M2 | 18.3808 | C2 | 18.3883 |
| M3 | 18.3967 | C3 | 18.4092 |
| M4 | 18.4033 | C4 | 18.4000 |
| M5 | 18.3900 | C5 | 18.3858 |
| M6 | 18.4108 | C6 | 18.3817 |
| A1 | 18.4033 | D1 | 18.3983 |
| A2 | 18.3933 | D2 | 18.3958 |
| A3 | 18.3992 | D3 | 18.3708 |
| A4 | 18.4067 | D4 | 18.4017 |
| A5 | 18.4067 | D5 | 18.4025 |
| A6 | 18.4025 | D6 | 18.3983 |
| Average value | 18.3967 | **Standard deviation** | 0.8652% |

**Supplementary Table 3.** Internal standard (L-2-chlorophenylalanine) retention time(plasma sample)

| Sample | R.T. (min) | Sample | R.T. (min) |
| --- | --- | --- | --- |
| N1 | 11.4867 | B1 | 11.4867 |
| N2 | 11.4867 | B2 | 11.4858 |
| N3 | 11.4867 | B3 | 11.4867 |
| N4 | 11.4867 | B4 | 11.4875 |
| N5 | 11.4875 | B5 | 11.4875 |
| N6 | 11.4875 | B6 | 11.4875 |
| M1 | 11.4925 | C1 | 11.4867 |
| M2 | 11.4858 | C2 | 11.4867 |
| M3 | 11.4867 | C3 | 11.4867 |
| M4 | 11.4867 | C4 | 11.4875 |
| M5 | 11.4875 | C5 | 11.4883 |
| M6 | 11.4875 | C6 | 11.4867 |
| A1 | 11.4867 | D1 | 11.4858 |
| A2 | 11.4867 | D2 | 11.4858 |
| A3 | 11.4867 | D3 | 11.4875 |
| A4 | 11.4867 | D4 | 11.4875 |
| A5 | 11.4875 | D5 | 11.4883 |
| A6 | 11.4867 | D6 | 11.4858 |
| Average value | 11.4870 | **Standard deviation** | 0.001133 |

**Supplementary Table 4.** Active compounds Information of Epimedium fried with suet oil

| Drug | Active compounds | Formula | CAS Number | Structural formula |
| --- | --- | --- | --- | --- |
| Epimedium | Quercetin | C_15_H_10_O_7_ | 117-39-5 | 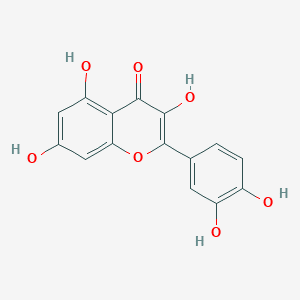 |
| Epimedium | Hexandraside E | C_32_H_38_O_16_ | 139955-75-2 | 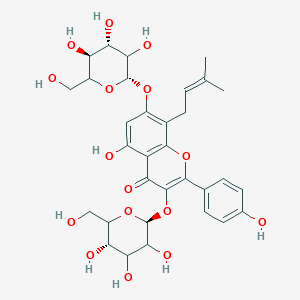 |
| Epimedium | Epimedoside E | C_37_H_46_O_19_ | NA | 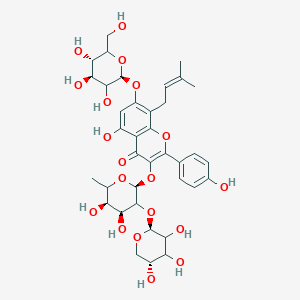 |
| Epimedium | Epimedin A | C_39_H_50_O_20_ | 110623-72-8 | 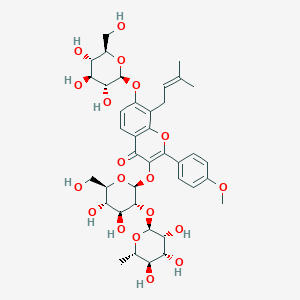 |
| Epimedium | Epimedin B | C_38_H_48_O_19_ | 110623-73-9 | 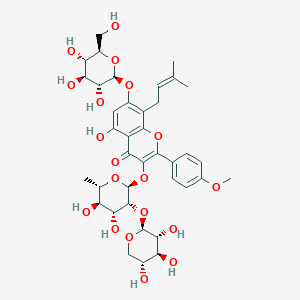 |
| Epimedium | Epimedin C | C_39_H_50_O_19_ | [110642-44-9](https://www.chemsrc.com/baike/951829.html) | 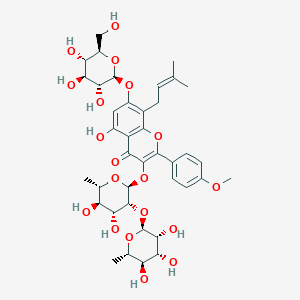 |
| Epimedium | Icariin | C_33_H_40_O_15_ | [489-32-7](https://www.chemsrc.com/baike/118539.html) | 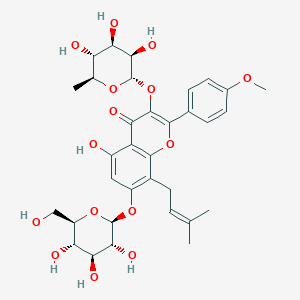 |
| Epimedium | Korepimedoside C/EpimedinⅠ | C_41_H_52_O_21_ | [205445-00-7](https://www.chemsrc.com/baike/1567895.html) | 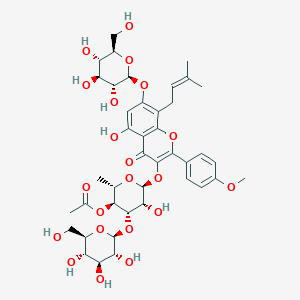 |
| Epimedium | Caohuoside B | C_45_H_56_O_23_ | NA | 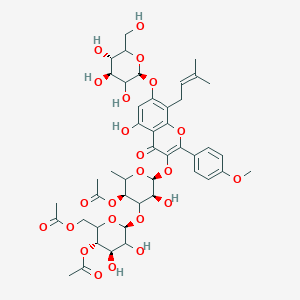 |
| Epimedium | Sagittatoside A | C_33_H_40_O_15_ | [118525-35-2](https://www.chemsrc.com/baike/649109.html) | 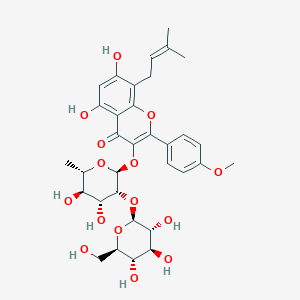 |
| Epimedium | Sagittatoside B | C_32_H_38_O_14_ | 118525-36-3 | 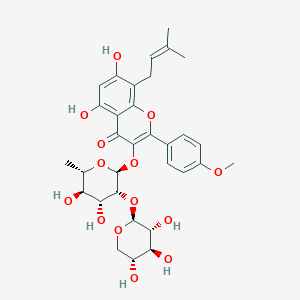 |
| Epimedium | Icariside II/Baohuoside I | C_27_H_30_O_10_ | 113558-15-9 | 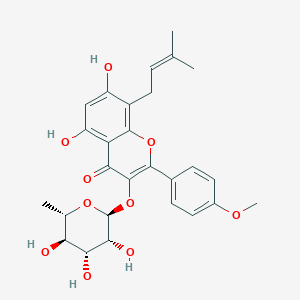 |
| Epimedium | 2''-O-Rhamnosylicariside II | C_33_H_40_O_14_ | 135293-13-9 | 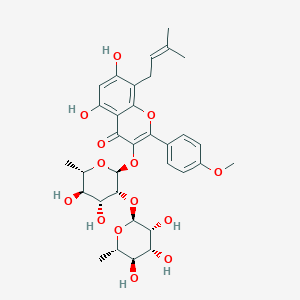 |
| Suet oil | Methyl dodecanoate | C_13_H_26_O_2_ | 111-82-0 | 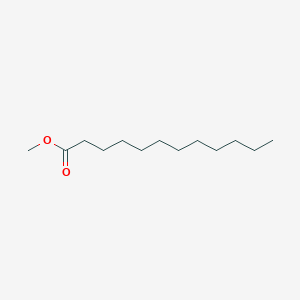 |
| Suet oil | Methyl (*Z*)-tetradec-9-enoate | C_15_H_28_O_2_ | 56219-06-8 | 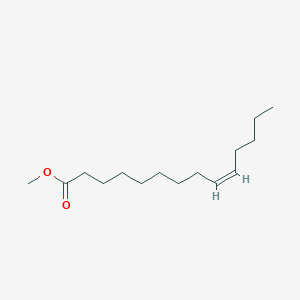 |
| Suet oil | Methyl 12-methyltridecanoate | C_15_H_30_O_2_ | 5129-58-8 | 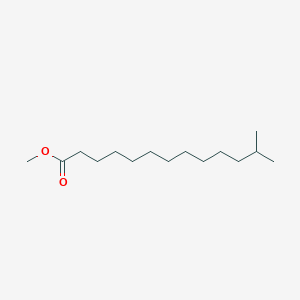 |
| Suet oil | Methyl tetradecanoate | C_15_H_30_O_2_ | 124-10-7 | 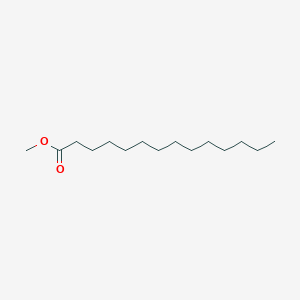 |
| Suet oil | Methyl pentadecanoate | C_16_H_32_O_2_ | 7132-64-1 | 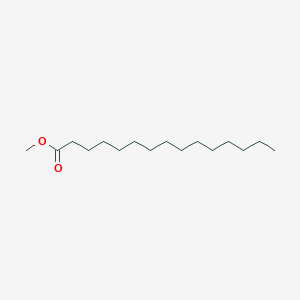 |
| Suet oil | Methyl (*Z*)-hexadec-9-enoate | C_17_H_32_O_2_ | 1120-25-8 | 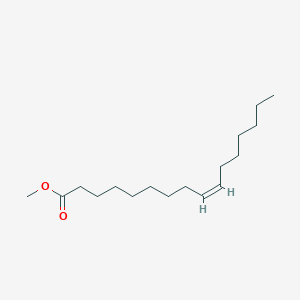 |
| Suet oil | Methyl hexadecanoate | C_17_H_34_O_2_ | 112-39-0 | 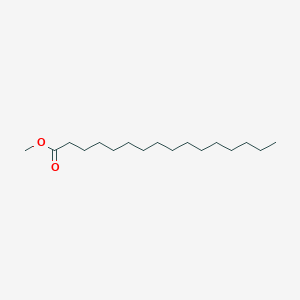 |
| Suet oil | Methyl 15-methylhexadecanoate | C_18_H_36_O_2_ | 6929-04-0 | 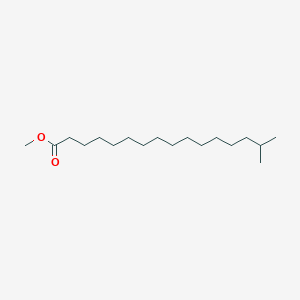 |
| Suet oil | Methyl (*Z*)-heptadec-10-enoate | C_18_H_34_O_2_ | 75190-82-8 | 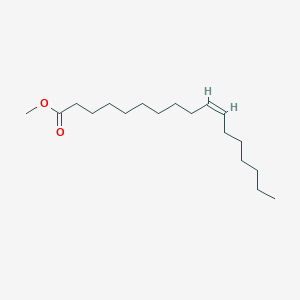 |
| Suet oil | Methyl heptadecanoate | C_18_H_36_O_2_ | 1731-92-6 | 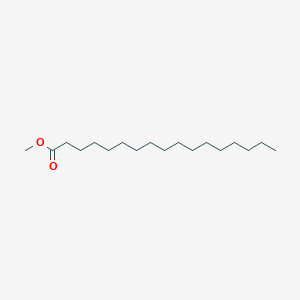 |
| Suet oil | Methyl linoleate | C_19_H_34_O_2_ | 112-63-0 | 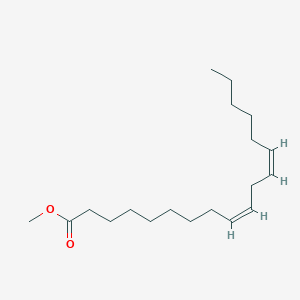 |
| Suet oil | Methyl (9*Z*,11*E*)-octadeca-9,11-dienoate | C_19_H_34_O_2_ | 13058-52-1 | 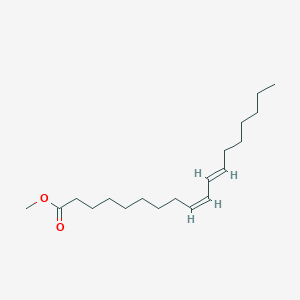 |
| Suet oil | Methyl (10*E*,12*Z*)-octadeca-10,12-dienoate | C_19_H_34_O_2_ | 21870-97-3 | 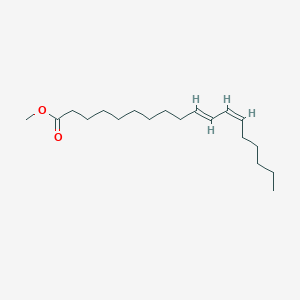 |
| Suet oil | Methyl (*E*)-octadec-9-enoate | C_19_H_36_O_2_ | 1937-62-8 | 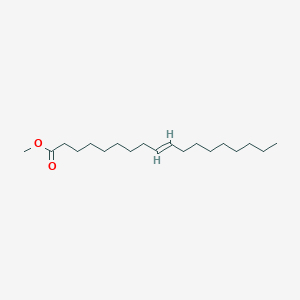 |
| Suet oil | Methyl oleate | C_19_H_36_O_2_ | 112-62-9 | 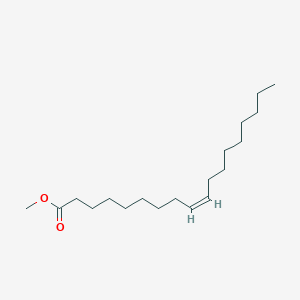 |
| Suet oil | Methyl (*E*)-octadec-11-enoate | C_19_H_36_O_2_ | 52380-33-3 | 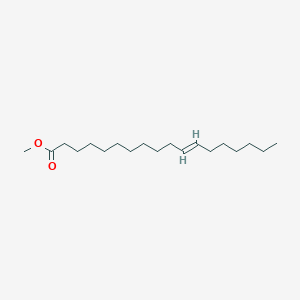 |
| Suet oil | Methyl octadecanoate | C_19_H_38_O_2_ | 112-61-8 | 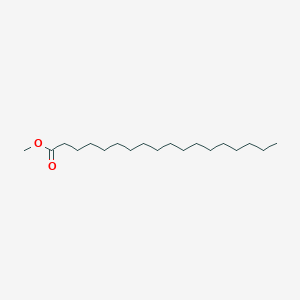 |
| Suet oil | Methyl (*Z*)-nonadec-10-enoate | C_20_H_38_O_2_ | 19788-74-0 | 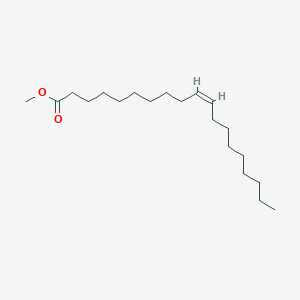 |
| Suet oil | Methyl (*E*)-nonadec-10-enoate | C_20_H_38_O_2_ | 56599-83-8 | 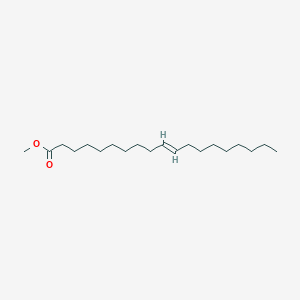 |
| Suet oil | Methyl 8-(2-octylcyclopropyl) octanoate | C_20_H_38_O_2_ | 10152-62-2 | 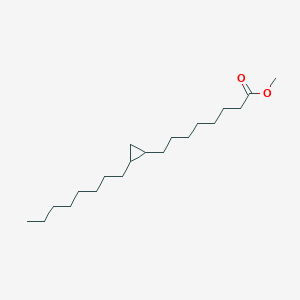 |
| Suet oil | Methyl nonadecanoate | C_20_H_40_O_2_ | 1731-94-8 | 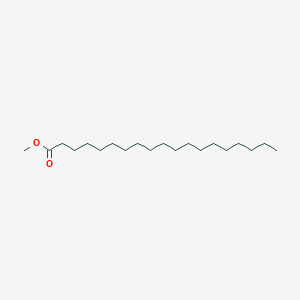 |
| Suet oil | Methyl (8*E*,11*E*,14*E*)-icosa-8,11,14-trienoate | C_21_H_36_O_2_ | 17364-32-8 | 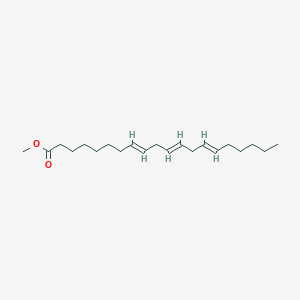 |
| Suet oil | Methyl (*Z*)-icos-11-enoate | C_21_H_40_O_2_ | 2390-09-2 | 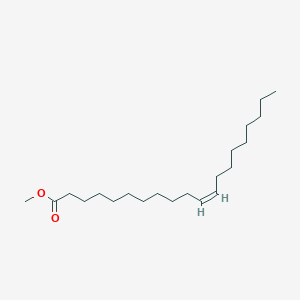 |
| Suet oil | Methyl icosanoate | C_21_H_42_O_2_ | 1120-28-1 | 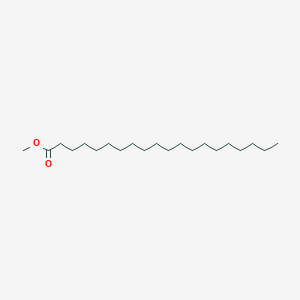 |

NA: No CAS number matched.
